# Host-pathogen interactome mapping for HTLV-1 and -2 retroviruses

**DOI:** 10.1186/1742-4690-9-26

**Published:** 2012-03-29

**Authors:** Nicolas Simonis, Jean-François Rual, Irma Lemmens, Mathieu Boxus, Tomoko Hirozane-Kishikawa, Jean-Stéphane Gatot, Amélie Dricot, Tong Hao, Didier Vertommen, Sébastien Legros, Sarah Daakour, Niels Klitgord, Maud Martin, Jean-François Willaert, Franck Dequiedt, Vincent Navratil, Michael E Cusick, Arsène Burny, Carine Van Lint, David E Hill, Jan Tavernier, Richard Kettmann, Marc Vidal, Jean-Claude Twizere

**Affiliations:** 1 Center for Cancer Systems Biology (CCSB) and Department of Cancer Biology, Dana-Farber Cancer Institute, 450 Brookline Ave., Boston, MA 02215, USA; 2 Department of Genetics, Harvard Medical School, 77 Avenue Louis Pasteur, Boston, MA 02115, USA; 3 Laboratoire de Bioinformatique des Génomes et des Réseaux (BiGRe), Université Libre de Bruxelles, Campus Plaine, CP 263, Boulevard du Triomphe, 1050 Bruxelles, Belgium; 4 Protein Signaling and Interactions - GIGA Research Center and Department of Chemistry - Gembloux ABT, University of Liège, 1 avenue de l'Hôpital, 4000 Liege, Belgium; 5 Department of Medical Protein Research, VIB, Ghent University, B-9000 Ghent, Belgium; 6 Laboratory of Molecular Virology, Institut de Biologie et de Médecine Moléculaires, Université Libre de Bruxelles, 12 Rue des Profs Jeener et Brachet, 6041 Gosselies, Belgium; 7 Hormones and Metabolism Unit, Université catholique de Louvain and de Duve Institute, 75 Avenue Hippocrate, 1200 Brussels, Belgium; 8 Ecole Nationale Vétérinaire de Lyon, Université de Lyon, INRA, UMR754, and INSERM, U851, 21 avenue Tony Garnier, Lyon F-69007, France

**Keywords:** HTLV, Interactome, Retrovirus, ORFeome, Tax, HBZ

## Abstract

**Background:**

Human T-cell leukemia virus type 1 (HTLV-1) and type 2 both target T lymphocytes, yet induce radically different phenotypic outcomes. HTLV-1 is a causative agent of Adult T-cell leukemia (ATL), whereas HTLV-2, highly similar to HTLV-1, causes no known overt disease. HTLV gene products are engaged in a dynamic struggle of activating and antagonistic interactions with host cells. Investigations focused on one or a few genes have identified several human factors interacting with HTLV viral proteins. Most of the available interaction data concern the highly investigated HTLV-1 Tax protein. Identifying shared and distinct host-pathogen protein interaction profiles for these two viruses would enlighten how they exploit distinctive or common strategies to subvert cellular pathways toward disease progression.

**Results:**

We employ a scalable methodology for the systematic mapping and comparison of pathogen-host protein interactions that includes stringent yeast two-hybrid screening and systematic retest, as well as two independent validations through an additional protein interaction detection method and a functional transactivation assay. The final data set contained 166 interactions between 10 viral proteins and 122 human proteins. Among the 166 interactions identified, 87 and 79 involved HTLV-1 and HTLV-2 -encoded proteins, respectively. Targets for HTLV-1 and HTLV-2 proteins implicate a diverse set of cellular processes including the ubiquitin-proteasome system, the apoptosis, different cancer pathways and the Notch signaling pathway.

**Conclusions:**

This study constitutes a first pass, with homogeneous data, at comparative analysis of host targets for HTLV-1 and -2 retroviruses, complements currently existing data for formulation of systems biology models of retroviral induced diseases and presents new insights on biological pathways involved in retroviral infection.

## Background

Human T-cell lymphotropic viruses HTLV-1 and -2 are members of *Deltaretrovirus *genus of the *Retroviridae *family [1]. HTLV-1 induces Adult T-cell Leukemia/Lymphoma (ATLL) [2], an aggressive lymphoproliferative disease. HTLV-1 is also associated with tropical spastic paraparesis (TSP) [3], a neurological degenerative syndrome. HTLV-2 is closely related to HTLV-1 but causes no known overt disease [4,5]. The elaborate pathogenicity of HTLV-1 involves establishment and reactivation of latent stages, transcriptional activation of specific cellular genes, and modulation of cell death and proliferation pathways [6]. Modulations of viral and cellular function upon infection rely on crosstalk between the few viral encoded proteins and specific human proteins.

HTLV genomes encode structural proteins that form the viral core particle (Gag and Env), and enzymatic retroviral proteins (reverse transcriptase, integrase and protease). HTLV contain a cluster of alternatively spliced open reading frames (ORFs) that encode regulatory proteins (Tax-1, Rex-1, HBZ, p30, p13, and p12 for HTLV-1 and Tax-2, Rex-2, APH-2, p28, p11 and p10 for HTLV-2).

Investigations focused on one or a few genes have identified numerous human factors interacting with HTLV viral proteins, with the results collected in several databases: *VirusMINT *[7] and *VirHostNet *[8]. Most of the available interaction data concern the highly investigated HTLV-1 Tax protein. Few protein-protein interactions (PPIs) have been reported for other HTLV-1 and HTLV-2 encoded proteins. Comparative molecular biology studies of HTLV-1 and HTLV-2 have focused primarily on the Tax oncoproteins [9,10]. Hence, many cellular proteins and pathways exploited by these retroviruses to induce disease are likely still unidentified. A systematic exploration of shared and distinct host-pathogen protein interaction profiles for these two viruses would likely identify novel molecular mechanisms linked to HTLV infection and be a useful tool for understanding how HTLV-1 subverts cellular pathways toward disease progression.

Our high-throughput yeast two-hybrid (HT-Y2H) technology employs well-defined collections of cloned open reading frames to provide systematic interrogation of potential PPIs [11-14]. HT-Y2H is amenable for investigating pathogen-host interactions [15,16]. Here, we adapted this strategy for the systematic mapping and comparison of pathogen-host PPIs. We report viral-host interactome maps for HTLV-1 and -2 retroviral proteomes with the human proteome; we compare the spectra of host targets for HTLV proteins and raise new hypotheses regarding the pathogenic activities of HTLV-1.

## Results and discussion

### Identification of HTLV - human protein interactions

To identify retroviral PPIs with the human proteome we adapted our well-established HT-Y2H system [12,14]. Using Gateway-based ORFeome libraries encoding HTLV-1 and HTLV-2 proteins (HTLV-1 Gag, Pol, Rex, Tax, Env, p12, p13, p30 and HTLV-2 Gag, Pol, Rex2, Tax2, Env and APH-2 - Additional file 1: Table S1) in a Y2H screen against the ~12,000 proteins expressed from Human ORFeome v3.1 [17], we identified 1028 diploid colonies representing 286 potential interactions between human proteins and HTLV viral proteins. These interactions were independently confirmed by pairwise Y2H retesting [12].

**Figure 1:**
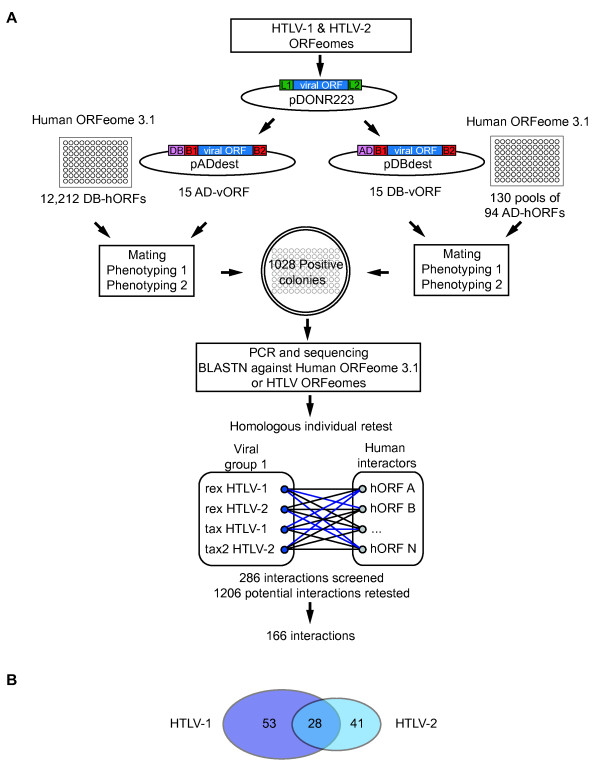
**Pipeline of the HT-Y2H experiment**. (**A**) Retroviral ORFeome screened against Human ORFeome 3.1 in both configurations (DB-hORF AD-rvORF and DB-rvORF AD-hORF). Interactions found in the primary screen were subjected to homologous individual retest, where any human interactor of HTLV 1-2 protein was also retested for interaction with all homologous HTLV l and 2 proteins. An example of homologous group with Tax and Rex is shown. To guarantee high specificity, only interactions identified with at least two out of three reporter phenotypes were considered positive. (**B**) Venn diagram of the number of human proteins targeted by each virus.

HTLV structural and regulatory proteins have significant sequence or functional similarity (for example HTLV-1 Tax and HTLV-2 Tax share 77% of sequence similarity, and both are transcriptional activators of viral expression). These homologous viral proteins might share one or more interacting partners amongst the human proteins, interactions that were not identified in initial screens because (i) highly overlapping or similar viral ORFs may be misidentified with BLAST, and (ii) interactions can be missed in a single screen [12,13,18]. We retested all homologous HTLV proteins for interaction with each human protein found in our initial screen with at least one homologous viral protein. For instance, all human proteins identified as HTLV-1 Tax interactors were also retested against HTLV-1 and HTLV-2 Tax and Rex proteins (Additional file 1: Table S1). This strategy combines the advantages of pooling [14] with individual testing, to reduce the cost and workload of the initial screen while keeping the ability to differentiate similar proteins, overcome sensitivity and specificity issues and permits comparison of negative results. The final data set contained 166 interactions between 10 viral proteins and 122 human proteins (Figure 1 and Additional file 1: Table S2). Among the 166 PPIs identified 87 and 79 interactions involved HTLV-1 and HTLV-2 -encoded proteins, respectively. Twenty-eight out of the one hundred and twenty-two human proteins were found to interact with both viruses (Figure 1B).

In addition to applying stringent internal controls and retests, to eliminate artifacts of the assay [19], we verified the quality of our HT-Y2H results by applying a binary interactome evaluation [12]. This evaluation employs independent protein-protein interaction assays to measure how any PPI dataset performs relative to a positive reference set (PRS) of high confidence manually curated interactions from the literature versus a random reference set (RRS) and position our dataset compared to these controls [12]. We tested 158 Y2H-identified binary interactions by mammalian protein-protein interaction trap assay (MAPPIT) [20]. MAPPIT is a forward mammalian two-hybrid strategy based on the activation of type I cytokine-signaling pathway. To perform a MAPPIT assay, we used as bait and prey, interacting partners fused to a STAT recruitment-deficient homodimeric cytokine receptor or to the C-terminal STAT3 recruitment portion of the gp130 receptor, respectively. Interactions between bait and prey proteins result in a functional cytokine receptor monitored by a STAT3-responsive promoter. The verification rate of our host-pathogen interactome data set by MAPPIT was 29% (40/137 testable pairs, Additional file 1: Table S2), which compares favorably to PRS detection rates [18]. As for other PPI assays tested so far, only a fraction of verifiable interactions detected by one PPI method will retest positive with another [18]. Previous studies show that MAPPIT detects about 20%-25% of PRS pairs under conditions that minimize the detection of RRS pairs [18]. As a control for specificity, a random set of 40 proteins from the human ORFeome 3.1 was also tested by MAPPIT for their interaction with HTLV proteins, and only 3 out of 40 (7.5%) were found positive. The MAPPIT retest rate of our HTLV-human PPIs represents ~80-100% of the maximum number of interactions expected to be recovered by MAPPIT, with an estimated false positive rate of 0-20% [12,13,18].

Human proteins interacting with viral proteins apparently have significantly different topological properties compared to random proteins in the human PPI network [15,21]. Viral proteins seem to preferentially target "hubs," i.e. highly connected proteins in the human-human PPI network. Preferential targeting of hubs is also observed in our HTLV network (Table 1). Human targets of HTLV proteins have higher connectivity (degree *k *= 13.75) compared to the whole network (*k *= 3.79), are more centrally located as measured by higher betweenness centrality (BC), 12443 for viral targets vs 2208 for random proteins, and have lower characteristic path length (CPL), 3.09 for viral targets vs. 4.38 for random proteins.

**Table 1:** Topological features of viral targets

	Viral Targets	Whole Network	P-value	Random Sources
Degree	13.75	3.79	2.53E-14	13.93

CPL	3.09	4.38	< 1E-320	3.07

Betweenness	12443	2208	6.14E-14	12646

Degree, Characteristic path length (CPL) and betweenness centrality (Betweenness) for the 131 human proteins identified in our screen (Viral Targets), the whole human PPI network (Whole Network), and for human proteins interacting with 19 random human proteins (Random Sources). P-values assess the difference between viral targets and the whole network

As previously demonstrated and again confirmed here, our Y2H methodology delivers high quality, reproducible biophysical interactions [12-14], but there is no guarantee that biophysical interactions are functionally relevant *in vivo*. To functionally validate our PPI dataset, we reasoned that some human proteins interacting with viral transactivators are likely to influence Tax transcriptional activities and thus contribute to viral replication and expression of cellular genes.

**Figure 2:**
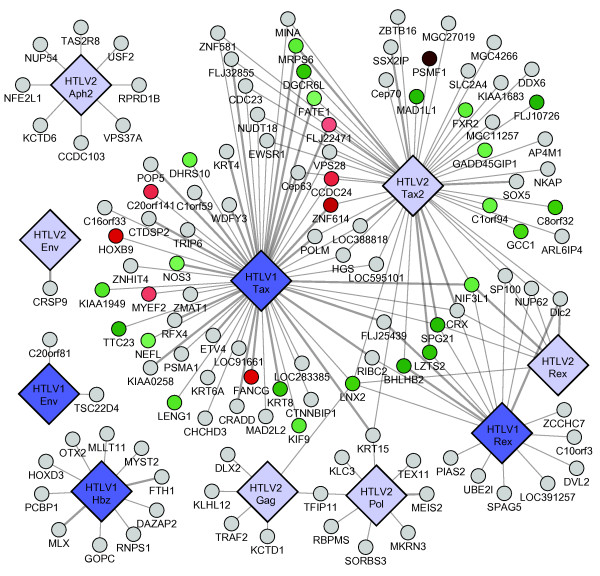
**HTLV 1 and 2 virus-host Y2H PPI network**. Big diamonds: viral ORFs, with HTLV-1 and HTLV-2 in blue and light blue, respectively. Small circles: Human ORFs, thin links: Y2H interactions; thick links: MAPPIT confirmed interactions. Human ORFs are colored according to transactivation assay results: green - down-regulation, red - up-regulation, dark red - stronger up-regulation, light green - weaker down-regulation.

**Figure 3:**
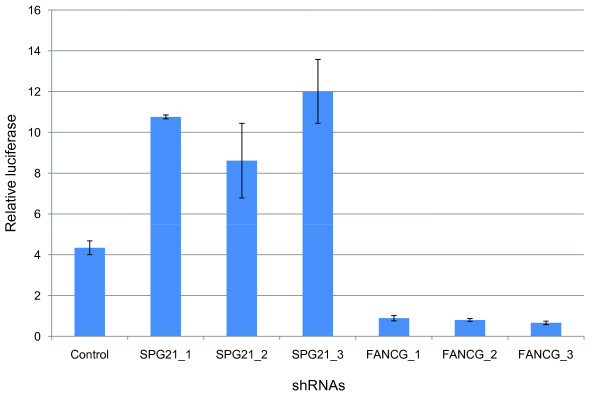
**Effect of SPG21 and FANCG knockdown on viral promoter activation**. Jurkat-LTR-Luc cells were transduced with lentiviral particles expressing a control shRNA and three validated shRNAs targeting various sequences of the SPG21 and FANCG mRNAs. Cells were cultured for 24 hours, and luciferase activities were determined from cell lysates and normalized to corresponding cell viability data (measured by WST1 test). Results are means of three experiments and error bars indicate standard errors.

Many HTLV-human interactions in our data set (106/166) involved the retroviral transactivator proteins HTLV-1 Tax (57/166) or HTLV-2 Tax2 (49/166). To examine the functional consequences of these associations, HEK293T cells were cotransfected with expression vectors for Tax-1 and Tax-interacting proteins, together with a firefly luciferase reporter driven by the HTLV-1 LTR promoter. As determined by normalized luciferase reporter assays, we identified 31 proteins (37% of the 83 Tax-interacting proteins) that regulated HTLV-1 LTR promoter activation by Tax (Figure 2 and Additional file 1: Table S3). There were 8 host factors that significantly enhanced Tax transactivation activities suggesting their potential implication in viral replication and persistence in infected cells. Another group of 23 cellular proteins down-regulated HTLV-1 LTR viral promoter activation and as such may be implicated in the viral latency which allows viruses to escape immune surveillance (Figure 2 and Additional file 1: Table S3). We selected two Tax1-cellular partners, SPG21, involved in the repression of T cell activation [22], and FANCG, a DNA damage response activated protein [23-25], for further validation in a T lymphocyte cell line. We used Jurkat T cells harboring a HTLV-1 LTR luciferase reporter (Jurkat-LTR-Luc) to confirm potential roles of SPG21 and FANCG in viral replication. We transduced Jurkat-LTR-Luc cells with a control shRNA and three validated shRNAs directed against SPG21 or FANCG and measured luciferase reporter-expression and cell viability. In accordance with regulation of Tax-transactivation data (Figure 2 and Additional file 1: Table S3), knockdown of SPG21 increased HTLV-1 LTR promoter activity while depletion of FANCG decreased HTLV-1 LTR promoter activity (Figure 3).

In summary, we identified 166 interactions between 10 viral proteins and 122 human proteins and verified their overall quality through an independent assay. We functionally validated our dataset by showing involvement of 31 human proteins in viral transcriptional regulation.

### Analysis of the HTLV-1 and-2 interactome maps

Our standardized experimental conditions, which combine stringent, high-throughput Y2H for a defined search space with systematic retesting of all homologous proteins, permit comparisons between interacting protein pairs. Network views of our data identify shared and distinct PPIs between HTLV-1 and HTLV-2. (Figure 2).

We found 34 human proteins that bind HTLV-1 Tax protein, but not the HTLV-2 Tax homolog (Figure 2 and Additional file 1: Table S4). Consistent with its intrinsically disordered conformation and pleiotropic activities [26], specific HTLV-1 Tax interactors include proteins associated with a range of distinct cellular functions such as transcription regulation (ETV4, RFX4, MyEF2, ZNHIT4, ZMAT1 and HOXB9), cell apoptosis (TRIP6 and CRADD), protein degradation (WDFY3 and PSMA1), and microtubule cytoskeleton (KIF9, KRT6A and KTR8).

We also found 26 HTLV-2 Tax interactors that did not interact with HTLV-1 Tax, including cell cycle proteins (Cep70, MAD1L1 and SSX2IP), transcription factors (NFKB activating protein, ZBTB16 and SOX5) and proteins involved in the endosomal-lysosomal system (AP4M1 and GCC1) (Figure 2 and Additional file 1: Table S4). Considering the differential oncogenic potential of the two HTLV viruses [9] and the central roles of their Tax proteins, these PPIs could shed light on mechanisms of cellular transformation by the Tax oncoprotein.

We have identified 10 novel HBZ binding proteins (Figure 2) including the homeobox transcription factor HOXD3; two RNA binding proteins, PCBP1 involved in restricting viral infections [27] and RNPS1, that can induce genomic instability when overexpressed [28]. Consistent with its association with transcriptional repression, we also found that HBZ interacts with MYST2, a member of the largest family of histone acetyltransferase enzymes, implicated in the regulation of DNA synthesis [29]. We also identified 8 novel APH-2 interactors (Figure 2 and Additional file 1: Table S2) including USF2, a member of the basic helix-loop-helix (bHLH) leucine zipper family of transcription factors that may play a role in late viral mRNA transcription [30]; VPS37A, a subunit of the mammalian endosomal sorting complex ESCRT-1 that have been shown to play a role in HIV-1 budding [31]; and NP54, a member of the nucleoporin complex that have been shown to bind HIV-1 Vpr and to play a critical role in the nucleocytoplasmic transport of viral preintegration complex [32]. Interestingly, we did not find any common interactor between HBZ and APH-2. The functions of these new HBZ and APH-2 associations with cellular factors remain to be further characterized.

### Comparison with known data

**Figure 4:**
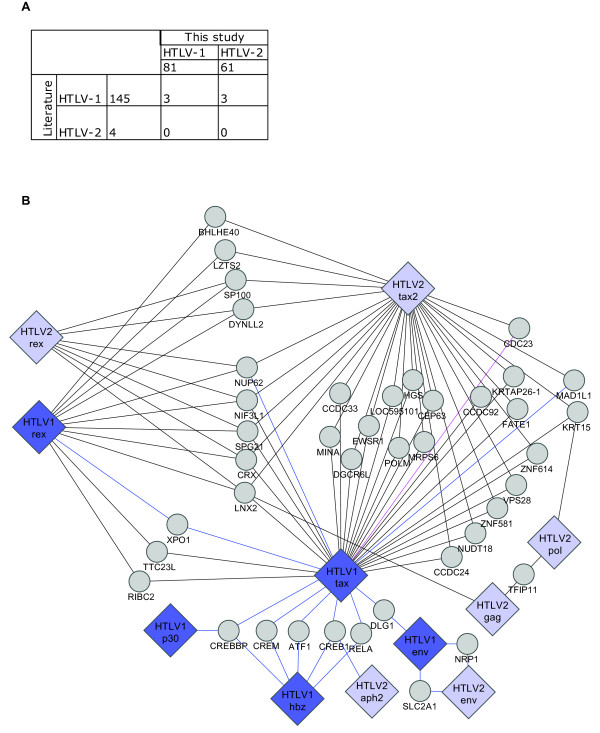
**Comparison to reported PPIs**. (**A**) Overlap between HTLV human targets curated from the literature (rows) and from our Y2H screen (columns). For each virus, the number directly below (columns) or beside (rows) the virus names gives the total number of human targets. The center shows the number of shared human targets between literature and our study. (**B**) Human proteins interacting with multiple viral proteins. Grey circles: human proteins, Blue edges: PPIs from literature curation; grey edges: Y2H PPIs found in our screen; magenta edge: PPIs found in our Y2H screen and literature curation.

Databases dedicated to virus-host PPIs (VirHostNet and VirusMint) contain only few PPI related to HTLV viruses. We thus manually curated the literature and found that most of host factors, which have been demonstrated to interact with HTLV proteins, concern the highly investigated HTLV-1 Tax (122/147) (Additional file 1: Table S5). The overlap between our study and known data is sparse (3 proteins: Nup62, MAD1L1 and Cdc23 - Figure 4A), not surprising given the use of dissimilar methods, clones, and search spaces. We integrated our dataset with current literature data on known human-HTLV PPIs and highlighted host factors interacting with at least two different viral proteins (Figure 4B). As examples, HTLV-1 HBZ, Tax and HTLV-2 APH-2 interact with CREB. Both HTLV-1 HBZ and Tax proteins interact with AP-1, CBP/p300, CREB, ATF and p65 NFκB transcription factors. However, interaction with these host factors drives opposite effects, as HBZ and APH-2 are involved in the repression of HTLV-transcription and are always expressed in leukemic cells [33,34].

### Enrichment of viral targets for biological pathways

The immediate human targets of HTLV proteins found here were not significantly enriched for annotated pathways in the Kyoto Encyclopedia of Genes and Genomes (KEGG) [35], i.e. the number of proteins belonging to a specific pathways is not significantly higher than random expectation, probably because of the limited number of human targets. To improve sensitivity, we also analyzed second-degree interactors, those human proteins in the human-human PPI network [14] that interact with human targets of viral proteins. Proteins associated with apoptotic pathways, Notch signaling, cell cycle, ubiquitin mediated proteolysis, as well as proteins involved in several human cancers including chronic myeloid leukemia, were overrepresented compared to random expectation (Table 2).

**Table 2:** KEGG pathways enriched in secondary viral interactors

Pathway Name	Pathway ID	Observed	Random	Odds Ratio	FDR	FDR-Corr
Apoptosis	hsa04210	6	1.82	3.31	1.3E-04	1.9E-02

Cell cycle	hsa04110	10	2.87	3.48	3.6E-04	4.8E-02

Chronic myeloid leukemia	hsa05220	8	1.59	5.03	2.1E-05	3.2E-03

Colorectal cancer	hsa05210	7	2.13	3.28	9.3E-05	1.4E-02

ErbB signaling pathway	hsa04012	7	1.67	4.19	6.3E-05	9.5E-03

Glioma	hsa05214	7	0.76	9.26	6.2E-05	9.3E-03

Huntington's disease	hsa05040	6	0.34	17.41	< 2.5E-06	< 3.8E-04

Insulin signaling pathway	hsa04910	10	2.06	4.86	1.2E-04	1.8E-02

Melanoma	hsa05218	4	0.64	6.27	1.4E-04	2.0E-02

Notch signaling pathway	hsa04330	4	1.82	2.20	< 2.5E-06	< 3.8E-04

Olfactory transduction	hsa04740	4	0.16	25.66	< 2.5E-06	< 3.8E-04

Prostate cancer	hsa05215	5	1.04	4.79	2.9E-04	4.3E-02

Ubiquitin mediated proteolysis	hsa04120	10	3.02	3.31	2.0E-04	2.9E-02

For each enriched KEGG pathway is given the pathway identifier in the KEGG database (Pathway ID), the number of observed proteins belonging to the considered pathway (Observed), the number of proteins in the pathway expected at random (Random), the ratio between the number of observed proteins and the expected number (Odds Ratio), the false discovery rate (FDR), and the corrected FDR (FDR-Corr)

### Apoptotic pathway

**Figure 5:**
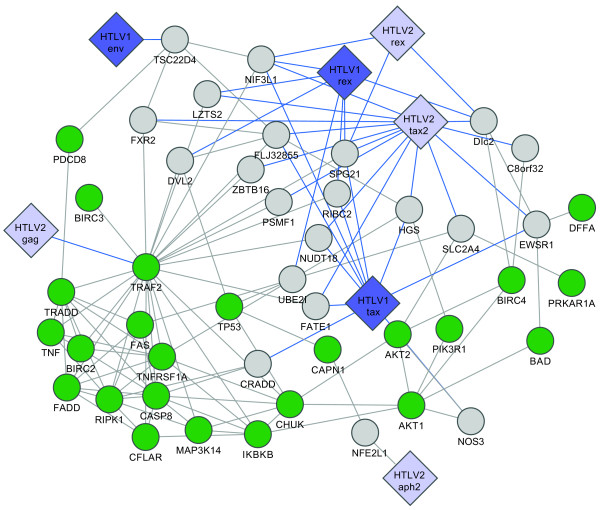
**Targeting of apoptotic pathway by viral proteins**. (**A**) Schematic representation of PPIs. Big diamonds: viral ORFs, with HTLV-1 and HTLV-2 in blue and light blue, respectively. Small circles: human ORFs with green representing membership of the apoptotic pathway. Grey links: human-human PPIs; blue links: virus-human PPIs.

**Figure 6:**
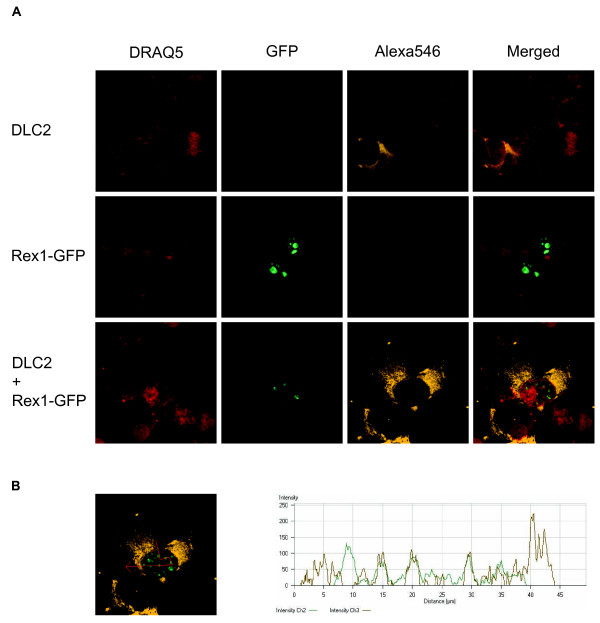
**HTLV-1 Rex and DLC2 co-localize in nucleolar foci**. (**A**) HeLa cells were transfected with expression vectors for Rex1-GFP and Flag-DLC2 as indicated. Twenty-four hours post-transfection, cells were labeled with anti-flag M2 mouse antibody followed by alexa546-conjugated anti-mouse secondary antibody. Cells were stained with the far-red DNA marker DRAQ5 and analyzed by confocal microscopy. Merge corresponds to the simultaneous acquisition of all three fluorochromes. (**B**) Fluorescent intensities were plotted along the red line segments. The green and orange lines in the profile correspond to the relative intensities of GFP and Alexa 546.

In an apoptotic pathway sub-network, KEGG analysis highlighted the tumor necrosis factor (TNF) receptor and the AKT/PI3K signaling pathways as potential targets for HTLV proteins. In this network HTLV Tax and Rex proteins are closely linked to the Akt/PI3K and mitochondrial apoptotic pathways. We identified interactions between HTLV Tax proteins and nitric oxide synthase 3 (NOS3), hepatocyte growth factor-regulated tyrosine kinase substrate (HGS), Ewing sarcoma breakpoint region 1 (EWSR1) and glucose transporter-4 (SLC2A4) proteins. KEGG analysis indicated that phosphatidylinositol-3-kinase (PI3K), BCL2-antagonist of cell death (Bad), and DNA fragmentation factor alpha (DFFA) proteins are second-degree targets of HTLV Tax proteins (Figure 5). We also found that the HTLV Rex proteins interact with DLC2 (for dynein light chain 2), able to regulate cell death-inducing functions of pro-apoptotic proteins Bim (Bcl-2-interacting mediator of cell death) and Bmf (Bcl-2-modifying factor). HTLV Rex proteins are nuclear-localizing proteins well known to drive post-transcriptional export of viral mRNAs from the nucleus to the cytoplasm [36-38]. Besides its interaction with the cellular export factor CRM1 [39], functional relationship between Rex proteins and their cellular partners have not been fully investigated. Interaction between Rex proteins and DLC2 may shed light on a new role of Rex in the apoptotic pathway. To assess the subcellular localization of Rex1 and DLC2, we transfected HeLa cells with expression vectors for Rex1-GFP and Flag-tagged DLC2. Cells were stained by anti-flag antibody followed by Alexa546-conjugated secondary antibody and a far-red fluorescent DNA dye (DRAQ5) for nuclear staining. Consistent with previous reports [40-42], DLC2 was found exclusively in the cytoplasm (Figure 6A, DLC2); and Rex-GFP was localized in nucleolar foci (Figure 6A, Rex1-GFP). Co-expression of Rex1-GFP and Flag-DLC2 provoked a change in the localization of DLC2 with two patterns being observed. DLC2 was localized in the cytoplasm as well as in nuclear foci (Figure 6A, DLC2 + Rex1-GFP, Alexa546). It thus appeared that coexpression with Rex1 directs DLC2 in nucleolar foci as revealed by the good match of the green (Rex1-GFP) and orange (Flag-DLC2) fluorochromes. We conclude that HTLV Rex proteins might interfere with the anti-apoptotic activities of DLC2 in HTLV infected cells.

**Figure 7:**
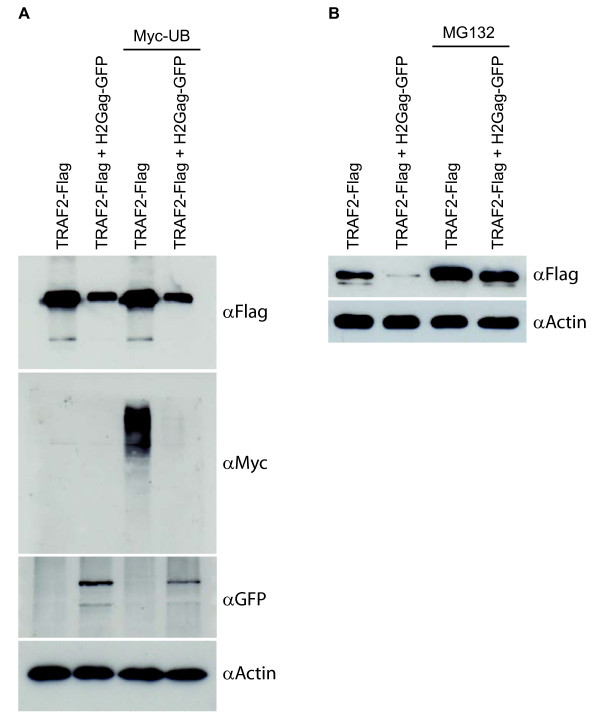
**Gag induces proteasomal degradation of TRAF2**. (**A**) Western blot of HEK293T cell extracts transfected with expressing vectors for Flag-TRAF2, HTLV-2Gag-GFP and Myc-ubiquitin. Cell extracts were immunoblotted with anti-Flag, anti-Myc, anti-GFP and anti-actin antibodies. (**B**) Western blot of HEK293T cells transfected with expressing vectors for Flag-TRAF2 and HTLV-2Gag-GFP, pre-treated or not with the proteasomal inhibitor MG-132 (1 μM) for 24 H. Cell extracts were immunoblotted with anti-Flag or anti-actin antibodies.

We also identified TNF receptor-associated factor type 2 (TRAF-2) as a central protein mediating interactions between HTLV proteins, TNF receptor (TNFR) signaling, and the Akt/PI3K survival pathway (Figure 5). We found that TRAF2 directly binds HTLV-2 Gag and is also a second-degree interactor of HTLV Tax and Rex proteins. Depending on its interacting partners, TRAF2 signals drive contradictory cellular responses. Direct binding to the cytoplasmic domain of TNFR2, which does not contain a death domain, can trigger NFκB and JNK activation, but TRAF2 also indirectly mediates the signal from a death domain containing receptors such as TNFR1 via interaction with FADD and TRADD pro-caspases adaptor factors [43]. Retroviral infection is frequently associated with elevated TNFα, and cell lines derived from ATL patients show sensitivity to TNF-related apoptosis [44]. Gag protein could target TRAF2 for proteasomal degradation, thereby facilitating sensitivity to TNFα-induced cell death. To investigate this possibility we co-expressed GFP tagged HTLV-2 Gag, Flag tagged TRAF2 and a Myc-Ubiquitin expressing vectors. The presence of HTLV-2 Gag reduced TRAF2 protein levels (Figure 7A, αFlag compare lanes 1 and 2; and lanes 3 and 4), and degradation of TRAF2 correlated with a reduction of Myc-ubiquitylated proteins (Figure 7A, αMyc compare lanes 3 and 4) suggesting that the TRAF2-E3 ubiquitin ligase activity was also affected by the presence of HTLV-2 Gag protein. The degradation of TRAF2 could be blocked by preincubating cells with proteasome inhibitor MG132 (Figure 7B). Together these data indicate that HTLV-2 Gag induces proteasomal degradation of TRAF2.

### Cell cycle

**Figure 8:**
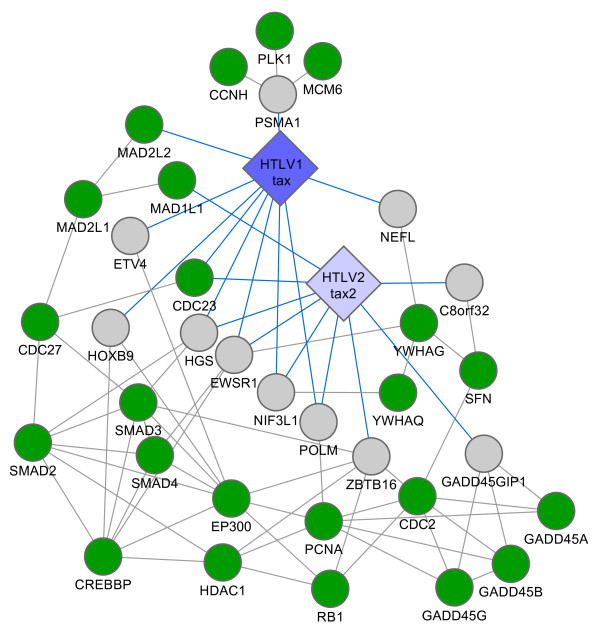
**Targeting of the cell cycle by viral proteins**. Schematic representation of PPIs. Big diamonds: viral ORFs, with HTLV-1 and HTLV-2 in blue and light blue, respectively. Small circles: human ORFs with green representing membership of the cell cycle. Grey links: human-human PPIs; blue links: virus-human PPIs.

Cell cycle is a tightly regulated cellular process targeted by transforming viruses to modulate cell division and proliferation. HTLV-1 Tax has been shown to bind cell cycle key regulators including cyclins-D1, D2 and D3, cyclin-dependent kinases (CDK) 4 and 6; and CDK inhibitor p16INK4a, to influence T lymphocyte G1-S progression [45-47]. HTLV-1 Tax also interacts with DNA repair and checkpoint proteins including checkpoint kinases (Chk) 1 and 2 and members of the mitotic spindle-assembly checkpoint (MAD1L1, MAD2L1 and MAD2L2) [48] (Figure 8). Common features in cell cycle regulation between HTLV-1 and -2 Tax proteins shown here, include their direct interaction with the MAD complex and with the anaphase-promoting complex or cyclosome (APC/C) via Cdc23 protein; and their indirect connection to similar cell cycle proteins such as Cdc27, Cdc2, PCNA and SMADs proteins (Figure 8). One difference highlighted here is the interaction of HTLV-1 Tax, and not HTLV-2 Tax, with the 26 proteasome subunit PSMA1, which could link HTLV-1 Tax to the minichromosome maintenance complex (MCM), the polo-like kinases (Plk) or the CDK-activating kinase complex (CCNH) (Figure 8). All these newly identified interactions should be validated in appropriate cell lines such as human hematopoietic stem cells (HSCs) previously used to demonstrate differences between Tax1 and 2 in cell cycle arrest in G0/G1 [9,49].

### Ubiquitin-mediated proteolysis pathway

**Figure 9:**
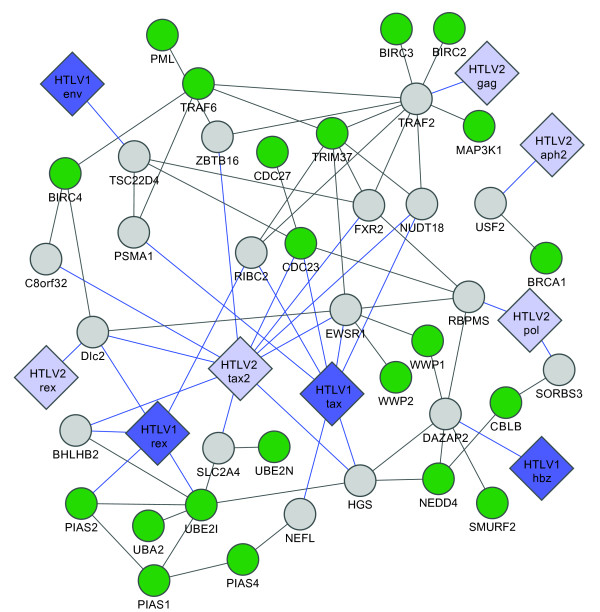
**Targeting of the Ubiquitin-mediated proteolysis pathway by viral proteins**. Schematic representation of PPIs. Big diamonds: viral ORFs, with HTLV-1 and HTLV-2 in blue and light blue, respectively. Small circles: human ORFs with green representing membership of the Ubiquitin-mediated proteolysis pathway. Grey links: human-human PPIs; blue links: virus-human PPIs.

We identified cellular E2 ubiquitin-conjugating enzymes UBE2I and UBE2N or UBC13; and E3 SUMO-protein ligases PIAS (protein inhibitor of activated STAT) 1, 2 and 4. Both types of enzymes have been previously shown to play a role in Tax-mediated NF-kB activation [50,51]. KEGG analysis also highlighted E3 ubiquitin ligases (CDC23, TRAF2 and TRAF6), which interact with HTLV proteins and which may play important roles in induced perturbations of the proteasomal pathway. CDC23 is a member of the anaphase promoting complex/cyclosome (APC/C, including CDC23), an E3 ubiquitin ligase that controls metaphase to anaphase transition [52-54]. TRAF proteins contain a RING finger domain, a domain that can simultaneously bind ubiquitination enzymes and their substrates [55,56] (Figure 9). HTLV-1 Tax might also provide a bridge to the proteasome by disrupting the interaction between an E3 ubiquitin ligase and its substrate, illustrated by the inactivation by Tax of the A20-Itch E3 ligase complex, potentially leading to a permanent activation of tumor necrosis factor (TNF) receptor (TNFR) signaling [57].

Most eukaryotic cellular proteins are selectively degraded by the ubiquitin-proteasome system [58]. Numerous infectious and cancer agents induce aberrations in the proteasomal pathway, and several inhibitors have been proposed as promising therapies [59-62]. Effective therapy faces challenges, as the activity of the proteasome is subjected to multiple regulation, and the selection of precise targeted proteins involves highly specific E2 and E3 ubiquitin enzymes [63].

### Notch pathway

**Figure 10:**
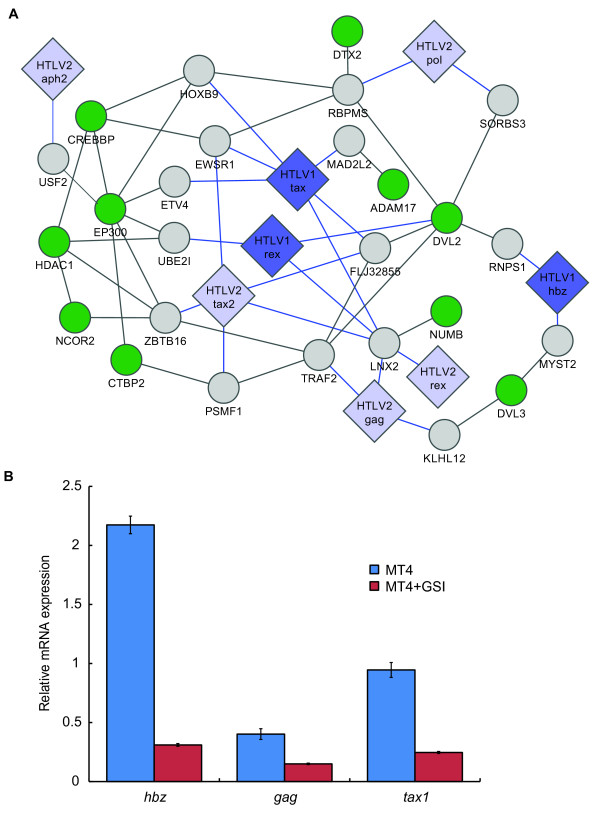
**Targeting of the Notch signalling pathway by viral proteins**. (**A**) Big diamonds: viral ORFs, with HTLV-1 and HTLV-2 in blue and light blue, respectively. Small circles: human ORFs with green representing membership of the Notch pathway. Grey links: human-human PPIs; blue links: virus-human PPIs. (**B**) Relative HTLV1-HBZ, -Gag and -Tax mRNA expression following MT4 cells treatment with or without 1 μM of γ-secretase inhibitor L685,458. Viral mRNA expression data are calculated relative to GAPDH mRNA expression data as 2^(CT(GAPDH)-CT(HBZ/Gag/Tax)) over three times triplicate experiments for each gene.

The highly conserved Notch signaling pathway regulates diverse cell fate decisions, including differentiation, proliferation, communication and specification. Several members of the Notch signaling pathway, including Numb [64], dishevelled (Dvl) proteins [65], cAMP-response element-binding protein (CREB)-binding protein (CREBBP or CBP) [66,67], and p300 [68], are targeted by HTLV Tax, Rex, Hbz, Gag and Pol proteins (Figure 10). It has been recently shown that the γ-secretase inhibitor (GSI) reduced tumor cell proliferation and tumor formation in an Adult T-cell Leukemia animal model [69]. To directly assess the involvement of the Notch pathway in viral infection, we treated an HTLV-1 transformed cell line (MT4) with a γ-secretase inhibitor (GSI) (L-685,458) [70] and tested whether inhibition of the Notch pathway could affect HTLV-1 expression in MT4 cell line. Interestingly, we showed by quantitative RT-PCR, that inhibition of the Notch pathway significantly lowered HTLV-1 HBZ (*p *< 2.1E-5), Gag (*p *< 0.04) and Tax1 (*p *< 0.003) expression in MT4 cells (Figure 10B), suggesting that GSI could be a new class of retroviral replication inhibitors.

## Conclusion

HTLV-1 and HTLV-2 are closely related human deltaretroviruses that have a similar genomic organization and share a high degree of sequence homology. Both viruses are able to immortalize T lymphocytes *in vitro*. In contrast to HTLV-1, HTLV-2 has not been conclusively associated with any known human disease. Most comparative studies to identify molecular differences between HTLV-1 and -2 are based on literature data on the viral encoded oncoproteins Tax-1 and Tax-2 activities (reviewed in [9,10])

Several global analyses of virus-host protein-protein interaction networks have led to interesting hypotheses about network topological properties and about shared target human proteins and pathways [8,21]. Such statistical analyses were done on collections of literature-curated information and thus are biased in several ways. Given an inherent 'inspection bias' some proteins are more heavily studied than others, with selection biased towards 'interesting' processes, diseases or potential applications, leading to a non-homogeneous representation of different viruses and proteins. Moreover, collections from public databases are constituted of a heterogeneous assortment of different assays, clones, variants, experimental conditions, or inferences. Comparing data obtained from different experiments severely limits the applicability of statistical analysis.

Here, we identified by a systematic stringent high-throughput methodology, cellular interacting partners for HTLV-1 Tax, Rex, Env and HBZ; and for HTLV-2 Tax, Rex, Env, Pol, Gag, and APH-2 (Figure 2 and Additional file 1: Table S2), providing the first attempt at a large scale comparative analysis of HTLV-1 and -2 host factors interactome with homogenous data. Although our data show several differences between HTLV-1 and -2 at the level of individual interactions with cellular targets, the findings do not show that they target distinct pathways. Cellular factors interacting with HTLV-1 and HTLV-2 seem to be involved in similar pathways (Apoptosis, Notch signaling, cell cycle, ubiquitin mediated proteolysis,...), but in different ways (Table 2 and Figures 5, 6, 7, 8, 9 and 10). This study identified many new host factors, raises new hypotheses and demonstrates the usefulness of the approach by experimental validation of some specific examples; but the incompleteness of the data does not allow us to build predictive models. Interactome maps presented here are incomplete for at least three reasons. First, the human ORFeome v3.1 collection we used covers only ~50% of the human proteome and does not include variants. Second, yeast two-hybrid, like any PPI assay, captures only a portion of protein-protein interactions [18]. Third, interactome screens are rarely conducted to saturation, i.e. yielding all possible interactions under the given conditions. To identify most physical interactions and to be able to build comprehensive systems biology models would require combining several assays, with each assay conducted to saturation, using the most complete collection of clones, including variants, and under a wide range of experimental conditions. In addition, all interactions should be functionally validated, localized and their dynamics studied. Current efforts to map protein-protein interactions should hopefully lead to near complete maps for several organisms in the future.

In conclusion, our experimental identification of 166 PPIs involving 10 HTLV-1 and -2 retroviral proteins with 122 human proteins extends and complements existing data on human-viral protein interactions [8,15,16,21,71,72].

We also identify and discuss common and distinct host cellular proteins targeted by HTLV-1 and -2 in relations with several cellular pathways, and we present innovative targets for further investigation of HTLV-induced network perturbations and illustrate the usefulness of this dataset by further investigation of Rex-DLC2, TRAF2-Gag and the involvement of the Notch pathway.

## Availability of supporting data

All protein-protein interaction data were submitted to VirHostNet http://pbildb1.univ-lyon1.fr/virhostnet. Interactions resulting from this study are provided in MIMIX specifications http://mibbi.org/index.php/Projects/MIMIx in Additional file 2.

## Methods

### Cloning of HTLV-1 and HTLV-2 ORFeomes

HTLV-1 and HTLV-2 ORFeomes were cloned by Gateway recombination methodology (*Invitrogen*) using as PCR templates the following DNA clones obtained through the AIDS Research and Reference Reagent Program, Division of AIDS, NIAID, NIH: MT-2, ATK, pH6 B 3.5 and pH6 B 5.0. DNA clones MT-2 [73], ATK [74], pH6 B 3.5 and pH6 B 5.0 from Dr. Irvin Chen [75,76]. Clones pcDNA-SP1 was obtained from Dr. Mesnard [77], Rex1-GFP from Dr. Bex [42] pSG5-APH2 from Dr. Mahieux [34], PcDNA-1-Tax1 from Dr. Bex [78] and BC20.2 from Dr. Green [79]. The specific primers for each ORF contained AttB1.1 and AttB2.1 Gateway recombination sites forward 5'GGGGACAACTTTGTACAAAAAAGTTGGC and reverse 5'GAGAGTTAGTGGCCCGCAGGTCGGGGGA, allowing recombinational cloning into the spectinomycin resistant donor vector pDONR223 by BP clonase (Invitrogen).

All full-length and partial retroviral ORFs (rvORFs) were transferred by LR cloning into pDB-dest and pAD-dest-CYH [19] to generate yeast expression vectors for DB-rvORF and AD-rvORF fusion proteins. The rvORFs were also transferred into Gateway MAPPIT vectors for the expression of chimeric bait and prey in mammalian cells [20]. For other functional assays, the human ORFs encoding proteins identified in Y2H experiments were transferred from their corresponding entry clones into pDEST-Flag destination vectors [80].

### High-throughput yeast two-hybrid

AD-rvORF and DB-rvORF yeast expressing vectors were transformed into two different *MAT***a **and *MAT*α strains of yeast, respectively: MaV103 and Y8800 for all AD-ORFs and MaV203 and Y8930 for all DB-ORFs. Transformed yeast cells were spotted on solid synthetic complete (Sc) media lacking tryptophan (Sc-T) to select for AD-rvORF clones, or lacking leucine (Sc-L) to select for DB-rvORF clones. Growing colonies were cultured in liquid Sc-L or Sc-T media and stored in glycerol for subsequent use. To eliminate autoactivator baits that activate reporter genes in the absence of AD plasmids, all DB-ORFs in Mav203 strain or Y8930 were individually tested for auto-activation by growth on solid SC-L-H medium containing 20 mM (Mav103 strain) or 2 mM (Y8930 strain) 3-amino-triazole (3-AT). Aliquots of AD-rvORF transformed yeast were pooled to generate the AD-rvORF library.

Yeast two-hybrid screening was as described [14]. Yeast matings were performed with Mav103 and MaV203 or with Y880 and Y8930. Each of 12,212 DB-ORFs *MAT*α yeast strains of the human ORFeome version 3.1 [17] was mated with a pool of *MAT***a **yeast strains containing individual retroviral AD-rvORFs. The screen was also done in the reciprocal orientation, mating individual retroviral DB-rvORF yeast clones with the 12,212 human AD-ORFs pooled into 65 mini-libraries [14]. Diploid cells were selected on solid media Sc-L-T-H (containing 20 mM 3-AT for the MaV strain), and *de novo *autoactivators were eliminated using the counter-selectable marker *CYH2 *[19]. Positive colonies were picked for PCR amplification and identification of interacting proteins by sequencing of the respective AD- and DB-ORFs.

Each human protein found to interact with viral proteins was individually retested against all homologous proteins in the HTLV viruses. To this end, we mated *MAT*α (Mav203 or Y8930) and *MAT***a **(Mav103 or Y8800) yeast cells containing individual DB and AD fused to interacting human and retroviral ORF, respectively. Resulting diploid cells were tested for activation of multiple reporter genes [14].

### MAPPIT assay

The mammalian protein-protein interaction trap (MAPPIT) [20] fuses a bait to a STAT recruitment-deficient, homodimeric cytokine receptor, while the prey is coupled to the C-terminal STAT recruitment portion of the gp130 receptor. HEK293T cells maintained in DMEM medium supplemented with 10% of fetal bovine serum, 2 mM glutamine, 100 U/ml of penicillin and streptomycin were cotransfected with a STAT-responsive luciferase reporter, the bait, and the prey or control constructs. Twenty-four hours post-transfection, cells were stimulated with erythropoietin or left untreated for an additional 24 hours. Luciferase activity was measured from two independent transfection experiments in triplicate. Each interaction pair was tested in both orientations. The "Experiment to Control Ratio" (ECR) was computed as the ratio of "bait + prey" (BP) signal over "bait + irrelevant prey" (BIP) or "prey + irrelevant bait" (PIB) signals. To account for the variability of the raw data, Fieller's confidence interval at 95% for the ratios BP/BIP and BP/PIB was computed from the raw induction values. Heterogeneous variances were assumed, using the test by Tamhane and Logan, inverted according to Fieller's theorem [81,82]. This test was run with the R statistical package 'pairwiseCI'. For a trial to be considered positive, the lower bound of the ECR confidence interval has to be > = 3 for both BP/BIP and BP/PIB ratios.

### Transactivation assay

The plasmid pHTLV1LTR-Luc, containing a luciferase reporter gene under the control of the HTLV-1 LTR promoter, a renilla luciferase control vector, and plasmids expressing HTLV-1 Tax and each human ORF found to interact with these viral proteins, were co-transfected into HEK293T cells by the calcium phosphate method. The LTR luciferase construct was obtained by subcloning HTLV-1 LTR promoter (a gift from F. Bex [78]) into pGL3-basic vector (promega). Twenty-four hours post-transfection, cells were washed three times with PBS, lysed, and relative luciferase activities determined from two independent transfection experiments in triplicate. We computed a paired *t*-test to assess the difference of the means between samples with and without the human interactor. For a trial to be considered positive, the relative luciferase activities have to be > = 2 or < = 0.5, and the p-value of the *t*-test < 0.05.

### Effect of SPG21 and FANCG knockdown on viral promoter activation

HTLV-1 LTR promoter fused to firefly luciferase was transduced into Jurkat cells using the pREP10 vector (Invitrogen). Selection with hygromycin B (100 μg/ml) was employed to obtain stable transfectants (Jurkat-LTR-Luc cells). Lentiviral particles expressing a control shRNA and validated shRNA targeting various sequences of the SPG21 and FANCG mRNAs [83] were prepared as described [84]. shRNAs were obtained from Sigma (TRCN0000300854, TRCN0000304152, TRCN0000304153, TRCN0000082858, TRCN0000082859, TRCN0000082860, TRC1). Infected Jurkat-LTR-Luc cells were selected using puromycin (10 μg/ml). Jurkat-LTR -Luc cells stably expressing shRNA for SPG21 (Jurkat-LTR-shSPG21_1 to 3 and Jurkat-LTR-FANCG_1 to 3) and control cells (Jurkat-LTR-luc expressing a sh control) were cultured for 24 hours, and luciferase activities were measured. An aliquot was used to assess cell viability using a WST1 kit as described by the manufacturer (Roche). Differences of expression were assessed with one-tailed Student's *t*-test on triplicate experiments.

### Topological analysis

We computed the mean degree, characteristic path length (CPL) and betweenness centrality in an unbiased human-human PPI network [14] for the 131 human proteins identified in the HT-Y2H screen. The CPL of a node (protein) is the mean of the shortest paths from all nodes to the considered node in the network. We used Mann-Whitney *U*-test to compare the degree, CPL and betweenness distributions of the 131 viral targets to the whole network.

### KEGG pathway analysis

Definitions of pathways came from the KEGG database (September 2008). We used Fisher's Exact Test to determine pathway enrichment of direct targets of viral proteins. To evaluate the significance of indirect targets enrichment, we ran 100,000 simulations where we randomized the identity of the direct targets. The interactors of these targets were identified in the unbiased PPI network [14]; interactors belonging to each pathway counted; and the resulting distribution compared to the observed counts. An empirical False Discovery Rate (FDR) determined the significance of the enrichment, with the FDR computed as the proportion of random trials giving at least the observed number of indirect targets in the considered pathway. The FDR was corrected for multiple testing using the Bonferroni correction. Pathways with a FDR Corr < 0.05 and at least four observed proteins were taken as significant.

To avoid study bias inherent to literature curation, we used the CCSB-HI1 network [14] to compute the enrichment of indirect targets for KEGG pathways. The plotted networks (Figures 5, 8, 9 and 10) were built from a literature-curated interaction (LCI) network to show the most complete information. The LCI network is the union of human PPIs from BIND [85], DIP [86], HPRD [87], INTACT [88], and MINT [89] interaction databases (April 2007).

To construct sub-networks (Figures 5, 8, 9 and 10) for each pathway, direct targets of viral proteins belonging to the corresponding KEGG pathway, and direct targets linked to viral proteins were selected as "seeds". Interactors of these seeds in the human-human LCI network and belonging to the considered pathway were then selected as indirect targets, and all interactions between seeds and indirects targets were plotted, along with our virus-human PPI network. All network figures were constructed with Cytoscape [90].

### Co-expression of TRAF2 and gag

HEK293T cells were cultured in a humidified atmosphere with 5% CO2 at 37°C in DMEM supplement with 10% of fetal bovine serum and antibiotics. HEK293T cells were transfected using the calcium phosphate method [91]. In some cases, cells were pretreated with proteasomal inhibitor MG132 for 24 hr; washed in ice-cold PBS and lysed in IPLS buffer (1% NP-40, 10% glycerol, 120 mM NaCl, 20 mM Tris pH 7.5, 2 mM EDTA, and complete protease inhibitor cocktail (Roche)). Cell lysates were analyzed by Western blot using an anti-Flag M2 (Sigma), anti-GFP (abcam) or anti-Myc (Santa Cruz Biotechnology) antibodies.

### Confocal microscopy

HeLa cells were transfected with expression vectors for Rex1-GFP and Flag tagged DLC2 using Lipofectamine 2000 according to the manufacturer instructions (Invitrogen). Twenty four hours post-transfection, cells were fixed in 4% formaldehyde (20 minutes at room temperature), permeabilized with 0.5% triton X-100, and incubated with anti Flag M2 antibody (Sigma) followed by Alexa546 -coupled anti-mouse secondary antibody (Invitrogen). After nuclear staining with DRAQ5 (Invitrogen), and fixing with mounting medium (fluoromount, Sigma), cells were analyzed using a Zeiss fluorescence confocal microscope (Carl Zeiss Microscopy).

### Inhibition of notch signaling

HTLV-1 transformed cell line (MT4) from Dr. Douglas Richman [92] was obtained through the AIDS Research and Reference Reagent Program, Division of AIDS, NIAID, NIH. MT4 cells were cultured in RPMI supplemented with 10% fetal bovine serum and antibiotics. MT-4 cells were treated for 48 hours with or without γ-secretase inhibitor (L-685,458) [70] at 1 μM. Total RNAs were then isolated by Trizol method, subjected to DNase treatment and cDNAs synthesized using the RevertAid First Strand cDNA Synthesis kit according to the manufacturer instructions (Fermentas). Quantitative real-time PCR for GAPDH, HBZ, Gag and Tax expression was on a StepOne instrument (Applied Biosystem) using SYBR green dye (Eurogentec). Viral mRNA expression data are calculated relative to GAPDH mRNA expression data as 2^(CT(GAPDH)-CT(HBZ/Gag/Tax)) over three times triplicate experiments for each gene, and differences were assessed through one-tailed Student's *t*-test.

## Abbreviations

HTLV-1: Human T-lymphotropic virus type-1; HTLV-2: Human T-lymphotropic virus type-2; HIV-1: Human immunodeficiency virus type 1; PPI: Protein-protein interaction; Y2H: Yeast two-hybrid; HT-Y2H: High-throughput yeast two-hybrid; MAPPIT: Mammalian protein-protein interaction trap; CPL: Characteristic path length; TNF: Tumor necrosis factor; FDR: False discovery rate.

## Competing interests

The authors declare that they have no competing interests.

## Authors' contributions

NS and J-CT designed and performed experiments, analyzed data and wrote the paper; J-FR and IL supervised some Y2H and MAPPIT experiments, respectively; TH and NK performed sequence analysis; THK, AD and J-FW performed some Y2H experiments; J-SG performed infection studies, MB performed confocal microscopy analysis, DV performed mass spectrometry analysis, SD, SL, MM, contributed to transactivation experiments and manuscript editing; VN curated PPI data. MEC, FD CVL and AB edited the manuscript; RK, MV, DEH and JT supervised the project. All authors read and approved the final manuscript.

## Supplementary Material

Additional file 1 **Table S1**. List of viral ORFs. **Table S2**. experimental results. **Table S3**. Host factors regulating HTLV-1 LTR promoter activation by Tax. **Table S4**. Human proteins interacting with Tax viral proteins. **Table S5**. List of HTLV-1 and -2 host factors extracted from public database (Virhosnet) or literature search. **Table S6**. Viral targets degrees HTLV_human_PPIs_MIMiX.txt PPIs experimental results - MIMiX standard. Click here for file

Additional file 2 **PPIs experimental results in MIMiX standard specifications**. Click here for file

## References

[B1] Poiesz BJ Ruscetti FW Gazdar AF Bunn PA Minna JD Gallo RC Detection and isolation of type C retrovirus particles from fresh and cultured lymphocytes of a patient with cutaneous T-cell lymphoma Proc Natl Acad Sci USA 1980 77 7415 7419 10.1073/pnas.77.12.7415 6261256 PMC350514

[B2] Hinuma Y Nagata K Hanaoka M Nakai M Matsumoto T Kinoshita KI Shirakawa S Miyoshi I Adult T-cell leukemia: antigen in an ATL cell line and detection of antibodies to the antigen in human sera Proc Natl Acad Sci USA 1981 78 6476 6480 10.1073/pnas.78.10.6476 7031654 PMC349062

[B3] Gessain A Barin F Vernant JC Gout O Maurs L Calender A de The G Antibodies to human T-lymphotropic virus type-I in patients with tropical spastic paraparesis Lancet 1985 2 407 410 2863442 10.1016/s0140-6736(85)92734-5

[B4] Kalyanaraman VS Sarngadharan MG Robert-Guroff M Miyoshi I Golde D Gallo RC A new subtype of human T-cell leukemia virus (HTLV-II) associated with a T-cell variant of hairy cell leukemia Science 1982 218 571 573 10.1126/science.6981847 6981847

[B5] Bartman MT Kaidarova Z Hirschkorn D Sacher RA Fridey J Garratty G Gibble J Smith JW Newman B Yeo AE Murphy EL Long-term increases in lymphocytes and platelets in human T-lymphotropic virus type II infection Blood 2008 112 3995 4002 10.1182/blood-2008-05-155960 18755983 PMC2581993

[B6] Kannian P Green PL Human T Lymphotropic Virus Type 1 (HTLV-1): Molecular Biology and Oncogenesis Viruses 2010 2 2037 2077 10.3390/v2092037 21994719 PMC3185741

[B7] Chatr-aryamontri A Ceol A Peluso D Nardozza A Panni S Sacco F Tinti M Smolyar A Castagnoli L Vidal M VirusMINT: a viral protein interaction database Nucleic Acids Res 2009 37 D669 D673 10.1093/nar/gkn739 18974184 PMC2686573

[B8] Navratil V de Chassey B Meyniel L Delmotte S Gautier C Andre P Lotteau V Rabourdin-Combe C VirHostNet: a knowledge base for the management and the analysis of proteome-wide virus-host interaction networks Nucleic Acids Res 2009 37 D661 D668 10.1093/nar/gkn794 18984613 PMC2686459

[B9] Feuer G Green PL Comparative biology of human T-cell lymphotropic virus type 1 (HTLV-1) and HTLV-2 Oncogene 2005 24 5996 6004 10.1038/sj.onc.1208971 16155606 PMC2659530

[B10] Bertazzoni U Turci M Avesani F Di Gennaro G Bidoia C Romanelli MG Intracellular localization and cellular factors interaction of HTLV-1 and HTLV-2 Tax proteins: similarities and functional differences Viruses 2011 3 541 560 10.3390/v3050541 21994745 PMC3185761

[B11] Yu H Braun P Yildirim MA Lemmens I Venkatesan K Sahalie J Hirozane-Kishikawa T Gebreab F Li N Simonis N High-quality binary protein interaction map of the yeast interactome network Science 2008 322 104 110 10.1126/science.1158684 18719252 PMC2746753

[B12] Venkatesan K Rual JF Vazquez A Stelzl U Lemmens I Hirozane-Kishikawa T Hao T Zenkner M Xin X Goh KI An empirical framework for binary interactome mapping Nat Methods 2009 6 83 90 10.1038/nmeth.1280 19060904 PMC2872561

[B13] Simonis N Rual JF Carvunis AR Tasan M Lemmens I Hirozane-Kishikawa T Hao T Sahalie JM Venkatesan K Gebreab F Empirically controlled mapping of the Caenorhabditis elegans protein-protein interactome network Nat Methods 2009 6 47 54 10.1038/nmeth.1279 19123269 PMC3057923

[B14] Rual JF Venkatesan K Hao T Hirozane-Kishikawa T Dricot A Li N Berriz GF Gibbons FD Dreze M Ayivi-Guedehoussou N Towards a proteome-scale map of the human protein-protein interaction network Nature 2005 437 1173 1178 10.1038/nature04209 16189514

[B15] Calderwood MA Venkatesan K Xing L Chase MR Vazquez A Holthaus AM Ewence AE Li N Hirozane-Kishikawa T Hill DE Epstein-Barr virus and virus human protein interaction maps Proc Natl Acad Sci USA 2007 104 7606 7611 10.1073/pnas.0702332104 17446270 PMC1863443

[B16] de Chassey B Navratil V Tafforeau L Hiet MS Aublin-Gex A Agaugue S Meiffren G Pradezynski F Faria BF Chantier T Hepatitis C virus infection protein network Mol Syst Biol 2008 4 230 18985028 10.1038/msb.2008.66 PMC2600670

[B17] Lamesch P Li N Milstein S Fan C Hao T Szabo G Hu Z Venkatesan K Bethel G Martin P hORFeome v3.1: a resource of human open reading frames representing over 10,000 human genes Genomics 2007 89 307 315 10.1016/j.ygeno.2006.11.012 17207965 PMC4647941

[B18] Braun P Tasan M Dreze M Barrios-Rodiles M Lemmens I Yu H Sahalie JM Murray RR Roncari L de Smet AS An experimentally derived confidence score for binary protein-protein interactions Nat Methods 2009 6 91 97 10.1038/nmeth.1281 19060903 PMC2976677

[B19] Vidalain PO Boxem M Ge H Li S Vidal M Increasing specificity in high-throughput yeast two-hybrid experiments Methods 2004 32 363 370 10.1016/j.ymeth.2003.10.001 15003598

[B20] Eyckerman S Verhee A der Heyden JV Lemmens I Ostade XV Vandekerckhove J Tavernier J Design and application of a cytokine-receptor-based interaction trap Nat Cell Biol 2001 3 1114 1119 10.1038/ncb1201-1114 11781573

[B21] Dyer MD Murali TM Sobral BW The landscape of human proteins interacting with viruses and other pathogens PLoS Pathog 2008 4 e32 10.1371/journal.ppat.0040032 18282095 PMC2242834

[B22] Zeitlmann L Sirim P Kremmer E Kolanus W Cloning of ACP33 as a novel intracellular ligand of CD4 J Biol Chem 2001 276 9123 9132 10.1074/jbc.M009270200 11113139

[B23] Huang M Kennedy R Ali AM Moreau LA Meetei AR D'Andrea AD Chen CC Human MutS and FANCM complexes function as redundant DNA damage sensors in the Fanconi Anemia pathway DNA Repair (Amst) 2011 10 1203 1212 10.1016/j.dnarep.2011.09.006 21975120

[B24] MacKay C Declais AC Lundin C Agostinho A Deans AJ MacArtney TJ Hofmann K Gartner A West SC Helleday T Identification of KIAA1018/FAN1, a DNA repair nuclease recruited to DNA damage by monoubiquitinated FANCD2 Cell 2010 142 65 76 10.1016/j.cell.2010.06.021 20603015 PMC3710700

[B25] Zhang P Sridharan D Lambert MW Knockdown of mu-calpain in Fanconi anemia, FA-A, cells by siRNA restores alphaII spectrin levels and corrects chromosomal instability and defective DNA interstrand cross-link repair Biochemistry 2010 49 5570 5581 10.1021/bi100656j 20518497 PMC2899890

[B26] Boxus M Twizere JC Legros S Dewulf JF Kettmann R Willems L The HTLV-1 Tax interactome Retrovirology 2008 5 76 10.1186/1742-4690-5-76 18702816 PMC2533353

[B27] Dinh PX Beura LK Panda D Das A Pattnaik AK Antagonistic effects of cellular poly(C) binding proteins on vesicular stomatitis virus gene expression J Virol 2011 85 9459 9471 10.1128/JVI.05179-11 21752917 PMC3165775

[B28] Li X Niu T Manley JL The RNA binding protein RNPS1 alleviates ASF/SF2 depletion-induced genomic instability RNA 2007 13 2108 2115 10.1261/rna.734407 17959926 PMC2080599

[B29] Thomas T Voss AK The diverse biological roles of MYST histone acetyltransferase family proteins Cell Cycle 2007 6 696 704 10.4161/cc.6.6.4013 17374998

[B30] Viollet B Lefrancois-Martinez AM Henrion A Kahn A Raymondjean M Martinez A Immunochemical characterization and transacting properties of upstream stimulatory factor isoforms J Biol Chem 1996 271 1405 1415 10.1074/jbc.271.3.1405 8576131

[B31] Stuchell MD Garrus JE Muller B Stray KM Ghaffarian S McKinnon R Krausslich HG Morham SG Sundquist WI The human endosomal sorting complex required for transport (ESCRT-I) and its role in HIV-1 budding J Biol Chem 2004 279 36059 36071 10.1074/jbc.M405226200 15218037

[B32] Popov S Rexach M Ratner L Blobel G Bukrinsky M Viral protein R regulates docking of the HIV-1 preintegration complex to the nuclear pore complex J Biol Chem 1998 273 13347 13352 10.1074/jbc.273.21.13347 9582382

[B33] Gaudray G Gachon F Basbous J Biard-Piechaczyk M Devaux C Mesnard JM The complementary strand of the human T-cell leukemia virus type 1 RNA genome encodes a bZIP transcription factor that down-regulates viral transcription J Virol 2002 76 12813 12822 10.1128/JVI.76.24.12813-12822.2002 12438606 PMC136662

[B34] Halin M Douceron E Clerc I Journo C Ko NL Landry S Murphy EL Gessain A Lemasson I Mesnard JM Human T-cell leukemia virus type 2 produces a spliced antisense transcript encoding a protein that lacks a classic bZIP domain but still inhibits Tax2-mediated transcription Blood 2009 114 2427 2438 10.1182/blood-2008-09-179879 19602711 PMC2746472

[B35] Kanehisa M Araki M Goto S Hattori M Hirakawa M Itoh M Katayama T Kawashima S Okuda S Tokimatsu T Yamanishi Y KEGG for linking genomes to life and the environment Nucleic Acids Res 2008 36 D480 D484 18077471 10.1093/nar/gkm882 PMC2238879

[B36] Ballaun C Farrington GK Dobrovnik M Rusche J Hauber J Bohnlein E Functional analysis of human T-cell leukemia virus type I rex-response element: direct RNA binding of Rex protein correlates with in vivo activity J Virol 1991 65 4408 4413 2072457 10.1128/jvi.65.8.4408-4413.1991 PMC248880

[B37] Kusuhara K Anderson M Pettiford SM Green PL Human T-cell leukemia virus type 2 Rex protein increases stability and promotes nuclear to cytoplasmic transport of gag/pol and env RNAs J Virol 1999 73 8112 8119 10482560 10.1128/jvi.73.10.8112-8119.1999 PMC112827

[B38] Kesic M Doueiri R Ward M Semmes OJ Green PL Phosphorylation regulates human T-cell leukemia virus type 1 Rex function Retrovirology 2009 6 105 10.1186/1742-4690-6-105 19919707 PMC2780990

[B39] Daelemans D Costes SV Lockett S Pavlakis GN Kinetic and molecular analysis of nuclear export factor CRM1 association with its cargo in vivo Mol Cell Biol 2005 25 728 739 10.1128/MCB.25.2.728-739.2005 15632073 PMC543413

[B40] Herzig RP Andersson U Scarpulla RC Dynein light chain interacts with NRF-1 and EWG, structurally and functionally related transcription factors from humans and drosophila J Cell Sci 2000 113 Pt 23 4263 4273 11069771 10.1242/jcs.113.23.4263

[B41] Shao Y Aplin AE ERK2 phosphorylation of serine 77 regulates Bmf pro-apoptotic activity Cell Death Dis 2012 3 e253 10.1038/cddis.2011.137 22258404 PMC3270271

[B42] Baydoun H Duc-Dodon M Lebrun S Gazzolo L Bex F Regulation of the human T-cell leukemia virus gene expression depends on the localization of regulatory proteins Tax, Rex and p30II in specific nuclear subdomains Gene 2007 386 191 201 10.1016/j.gene.2006.09.008 17071021

[B43] Hsu H Shu HB Pan MG Goeddel DV TRADD-TRAF2 and TRADD-FADD interactions define two distinct TNF receptor 1 signal transduction pathways Cell 1996 84 299 308 10.1016/S0092-8674(00)80984-8 8565075

[B44] Bleumink M Kohler R Giaisi M Proksch P Krammer PH Li-Weber M Rocaglamide breaks TRAIL resistance in HTLV-1-associated adult T-cell leukemia/lymphoma by translational suppression of c-FLIP expression Cell Death Differ 2011 18 362 370 10.1038/cdd.2010.99 20706274 PMC3131883

[B45] Haller K Wu Y Derow E Schmitt I Jeang KT Grassmann R Physical interaction of human T-cell leukemia virus type 1 Tax with cyclin-dependent kinase 4 stimulates the phosphorylation of retinoblastoma protein Mol Cell Biol 2002 22 3327 3338 10.1128/MCB.22.10.3327-3338.2002 11971966 PMC133776

[B46] Suzuki T Kitao S Matsushime H Yoshida M HTLV-1 Tax protein interacts with cyclin-dependent kinase inhibitor p16INK4A and counteracts its inhibitory activity towards CDK4 EMBO J 1996 15 1607 1614 8612584 PMC450070

[B47] Iwanaga R Ohtani K Hayashi T Nakamura M Molecular mechanism of cell cycle progression induced by the oncogene product Tax of human T-cell leukemia virus type I Oncogene 2001 20 2055 2067 10.1038/sj.onc.1204304 11360190

[B48] Jin DY Spencer F Jeang KT Human T cell leukemia virus type 1 oncoprotein Tax targets the human mitotic checkpoint protein MAD1 Cell 1998 93 81 91 10.1016/S0092-8674(00)81148-4 9546394

[B49] Tripp A Liu Y Sieburg M Montalbano J Wrzesinski S Feuer G Human T-cell leukemia virus type 1 tax oncoprotein suppression of multilineage hematopoiesis of CD34+ cells in vitro J Virol 2003 77 12152 12164 10.1128/JVI.77.22.12152-12164.2003 14581552 PMC254283

[B50] Shembade N Harhaj NS Yamamoto M Akira S Harhaj EW The human T-cell leukemia virus type 1 Tax oncoprotein requires the ubiquitin-conjugating enzyme Ubc13 for NF-kappaB activation J Virol 2007 81 13735 13742 10.1128/JVI.01790-07 17942533 PMC2168884

[B51] Kfoury Y Setterblad N El-Sabban M Zamborlini A Dassouki Z El Hajj H Hermine O Pique C de The H Saib A Bazarbachi A Tax ubiquitylation and SUMOylation control the dynamic shuttling of Tax and NEMO between Ubc9 nuclear bodies and the centrosome Blood 2011 117 190 199 10.1182/blood-2010-05-285742 20959607

[B52] Merling R Chen C Hong S Zhang L Liu M Kuo YL Giam CZ HTLV-1 Tax mutants that do not induce G1 arrest are disabled in activating the anaphase promoting complex Retrovirology 2007 4 35 10.1186/1742-4690-4-35 17535428 PMC1894815

[B53] Liu B Hong S Tang Z Yu H Giam CZ HTLV-I Tax directly binds the Cdc20-associated anaphase-promoting complex and activates it ahead of schedule Proc Natl Acad Sci USA 2005 102 63 68 10.1073/pnas.0406424101 15623561 PMC544051

[B54] Kuo YL Giam CZ Activation of the anaphase promoting complex by HTLV-1 tax leads to senescence EMBO J 2006 25 1741 1752 10.1038/sj.emboj.7601054 16601696 PMC1440834

[B55] Lorick KL Jensen JP Fang S Ong AM Hatakeyama S Weissman AM RING fingers mediate ubiquitin-conjugating enzyme (E2)-dependent ubiquitination Proc Natl Acad Sci USA 1999 96 11364 11369 10.1073/pnas.96.20.11364 10500182 PMC18039

[B56] Joazeiro CA Weissman AM RING finger proteins: mediators of ubiquitin ligase activity Cell 2000 102 549 552 10.1016/S0092-8674(00)00077-5 11007473

[B57] Shembade N Harhaj NS Parvatiyar K Copeland NG Jenkins NA Matesic LE Harhaj EW The E3 ligase Itch negatively regulates inflammatory signaling pathways by controlling the function of the ubiquitin-editing enzyme A20 Nat Immunol 2008 9 254 262 10.1038/ni1563 18246070

[B58] Glickman MH Ciechanover A The ubiquitin-proteasome proteolytic pathway: destruction for the sake of construction Physiol Rev 2002 82 373 428 11917093 10.1152/physrev.00027.2001

[B59] Chellappan S Kraus VB Kroger B Munger K Howley PM Phelps WC Nevins JR Adenovirus E1A, simian virus 40 tumor antigen, and human papillomavirus E7 protein share the capacity to disrupt the interaction between transcription factor E2F and the retinoblastoma gene product Proc Natl Acad Sci USA 1992 89 4549 4553 10.1073/pnas.89.10.4549 1316611 PMC49120

[B60] Kalejta RF Shenk T Proteasome-dependent, ubiquitin-independent degradation of the Rb family of tumor suppressors by the human cytomegalovirus pp 71 protein Proc Natl Acad Sci USA 2003 100 3263 3268 10.1073/pnas.0538058100 12626766 PMC152280

[B61] Wang J Sampath A Raychaudhuri P Bagchi S Both Rb and E7 are regulated by the ubiquitin proteasome pathway in HPV-containing cervical tumor cells Oncogene 2001 20 4740 4749 10.1038/sj.onc.1204655 11498796

[B62] Soucy TA Smith PG Milhollen MA Berger AJ Gavin JM Adhikari S Brownell JE Burke KE Cardin DP Critchley S An inhibitor of NEDD8-activating enzyme as a new approach to treat cancer Nature 2009 458 732 736 10.1038/nature07884 19360080

[B63] Pickart CM Mechanisms underlying ubiquitination Annu Rev Biochem 2001 70 503 533 10.1146/annurev.biochem.70.1.503 11395416

[B64] Zhong W Jiang MM Weinmaster G Jan LY Jan YN Differential expression of mammalian Numb, Numblike and Notch1 suggests distinct roles during mouse cortical neurogenesis Development 1997 124 1887 1897 9169836 10.1242/dev.124.10.1887

[B65] Cotter D Honavar M Lovestone S Raymond L Kerwin R Anderton B Everall I Disturbance of Notch-1 and Wnt signalling proteins in neuroglial balloon cells and abnormal large neurons in focal cortical dysplasia in human cortex Acta Neuropathol 1999 98 465 472 10.1007/s004010051111 10541869

[B66] Kang-Decker N Tong C Boussouar F Baker DJ Xu W Leontovich AA Taylor WR Brindle PK van Deursen JM Loss of CBP causes T cell lymphomagenesis in synergy with p27Kip1 insufficiency Cancer Cell 2004 5 177 189 10.1016/S1535-6108(04)00022-4 14998493

[B67] Teo JL Ma H Nguyen C Lam C Kahn M Specific inhibition of CBP/beta-catenin interaction rescues defects in neuronal differentiation caused by a presenilin-1 mutation Proc Natl Acad Sci USA 2005 102 12171 12176 10.1073/pnas.0504600102 16093313 PMC1189325

[B68] Maekawa Y Minato Y Ishifune C Kurihara T Kitamura A Kojima H Yagita H Sakata-Yanagimoto M Saito T Taniuchi I Notch2 integrates signaling by the transcription factors RBP-J and CREB1 to promote T cell cytotoxicity Nat Immunol 2008 9 1140 1147 10.1038/ni.1649 18724371

[B69] Pancewicz J Taylor JM Datta A Baydoun HH Waldmann TA Hermine O Nicot C Notch signaling contributes to proliferation and tumor formation of human T-cell leukemia virus type 1-associated adult T-cell leukemia Proc Natl Acad Sci USA 2010 107 38 16619 16624 Epub 2010 Sep 7 10.1073/pnas.1010722107 20823234 PMC2944748

[B70] Shearman MS Beher D Clarke EE Lewis HD Harrison T Hunt P Nadin A Smith AL Stevenson G Castro JL L-685,458, an aspartyl protease transition state mimic, is a potent inhibitor of amyloid beta-protein precursor gamma-secretase activity Biochemistry 2000 39 8698 8704 10.1021/bi0005456 10913280

[B71] Ptak RG Fu W Sanders-Beer BE Dickerson JE Pinney JW Robertson DL Rozanov MN Katz KS Maglott DR Pruitt KD Dieffenbach CW Cataloguing the HIV type 1 human protein interaction network AIDS Res Hum Retroviruses 2008 24 1497 1502 10.1089/aid.2008.0113 19025396 PMC2655106

[B72] Uetz P Dong YA Zeretzke C Atzler C Baiker A Berger B Rajagopala SV Roupelieva M Rose D Fossum E Haas J Herpesviral protein networks and their interaction with the human proteome Science 2006 311 239 242 10.1126/science.1116804 16339411

[B73] Gray GS White M Bartman T Mann D Envelope gene sequence of HTLV-1 isolate MT-2 and its comparison with other HTLV-1 isolates Virology 1990 177 391 395 10.1016/0042-6822(90)90498-G 2353464

[B74] Seiki M Hattori S Hirayama Y Yoshida M Human adult T-cell leukemia virus: complete nucleotide sequence of the provirus genome integrated in leukemia cell DNA Proc Natl Acad Sci USA 1983 80 3618 3622 10.1073/pnas.80.12.3618 6304725 PMC394101

[B75] Chen IS McLaughlin J Gasson JC Clark SC Golde DW Molecular characterization of genome of a novel human T-cell leukaemia virus Nature 1983 305 502 505 10.1038/305502a0 6312323

[B76] Shimotohno K Wachsman W Takahashi Y Golde DW Miwa M Sugimura T Chen IS Nucleotide sequence of the 3' region of an infectious human T-cell leukemia virus type II genome Proc Natl Acad Sci USA 1984 81 6657 6661 10.1073/pnas.81.21.6657 6093110 PMC391989

[B77] Cavanagh MH Landry S Audet B Arpin-Andre C Hivin P Pare ME Thete J Wattel E Marriott SJ Mesnard JM Barbeau B HTLV-I antisense transcripts initiating in the 3'LTR are alternatively spliced and polyadenylated Retrovirology 2006 3 15 10.1186/1742-4690-3-15 16512901 PMC1459196

[B78] Bex F McDowall A Burny A Gaynor R The human T-cell leukemia virus type 1 transactivator protein Tax colocalizes in unique nuclear structures with NF-kappaB proteins J Virol 1997 71 3484 3497 9094620 10.1128/jvi.71.5.3484-3497.1997 PMC191495

[B79] Green PL Ross TM Chen IS Pettiford S Human T-cell leukemia virus type II nucleotide sequences between env and the last exon of tax/rex are not required for viral replication or cellular transformation J Virol 1995 69 387 394 7983733 10.1128/jvi.69.1.387-394.1995 PMC188586

[B80] Barrios-Rodiles M Brown KR Ozdamar B Bose R Liu Z Donovan RS Shinjo F Liu Y Dembowy J Taylor IW High-throughput mapping of a dynamic signaling network in mammalian cells Science 2005 307 1621 1625 10.1126/science.1105776 15761153

[B81] Tamhane AC Logan BR Finding the maximum safe dose level for heteroscedastic data J Biopharm Stat 2004 14 843 856 10.1081/BIP-200035413 15587967

[B82] Hasler M Vonk R Hothorn LA Assessing non-inferiority of a new treatment in a three-arm trial in the presence of heteroscedasticity Stat Med 2008 27 490 503 10.1002/sim.3052 17853384

[B83] Root DE Hacohen N Hahn WC Lander ES Sabatini DM Genome-scale loss-of-function screening with a lentiviral RNAi library Nat Methods 2006 3 715 719 10.1038/nmeth924 16929317

[B84] Tiscornia G Singer O Verma IM Production and purification of lentiviral vectors Nat Protoc 2006 1 241 245 10.1038/nprot.2006.37 17406239

[B85] Bader GD Betel D Hogue CW BIND: the Biomolecular Interaction Network Database Nucleic Acids Res 2003 31 248 250 10.1093/nar/gkg056 12519993 PMC165503

[B86] Xenarios I Salwinski L Duan XJ Higney P Kim SM Eisenberg D DIP, the Database of Interacting Proteins: a research tool for studying cellular networks of protein interactions Nucleic Acids Res 2002 30 303 305 10.1093/nar/30.1.303 11752321 PMC99070

[B87] Mishra GR Suresh M Kumaran K Kannabiran N Suresh S Bala P Shivakumar K Anuradha N Reddy R Raghavan TM Human protein reference database-2006 update Nucleic Acids Res 2006 34 D411 D414 10.1093/nar/gkj141 16381900 PMC1347503

[B88] Kerrien S Alam-Faruque Y Aranda B Bancarz I Bridge A Derow C Dimmer E Feuermann M Friedrichsen A Huntley R IntAct-open source resource for molecular interaction data Nucleic Acids Res 2007 35 D561 D565 10.1093/nar/gkl958 17145710 PMC1751531

[B89] Chatr-aryamontri A Ceol A Palazzi LM Nardelli G Schneider MV Castagnoli L Cesareni G MINT: the Molecular INTeraction database Nucleic Acids Res 2007 35 D572 D574 10.1093/nar/gkl950 17135203 PMC1751541

[B90] Yeung N Cline MS Kuchinsky A Smoot ME Bader GD Exploring biological networks with Cytoscape software Curr Protoc Bioinformatics 2008 Chapter 8 Unit 8 13 10.1002/0471250953.bi0813s23 18819078

[B91] Twizere JC Springael JY Boxus M Burny A Dequiedt F Dewulf JF Duchateau J Portetelle D Urbain P Van Lint C Human T-cell leukemia virus type-1 Tax oncoprotein regulates G-protein signaling Blood 2007 109 1051 1060 16990599 10.1182/blood-2006-06-026781 PMC1785145

[B92] Harada S Koyanagi Y Yamamoto N Infection of HTLV-III/LAV in HTLV-I-carrying cells MT-2 and MT-4 and application in a plaque assay Science 1985 229 563 566 10.1126/science.2992081 2992081

