# Supplementary material for: Host-pathogen interactome mapping for HTLV-1 and -2 retroviruses
Source: Retrovirology. 2012 Mar 29;9:26. doi: 10.1186/1742-4690-9-26 (PMC3351729; doi:10.1186/1742-4690-9-26)
Supplement: Additional file 1 — Table S1. List of viral ORFs. Table S2. experimental results. Table S3. Host factors regulating HTLV-1 LTR promoter activation by Tax. Table S4. Human proteins interacting with Tax viral proteins. Table S5. List of HTLV-1 and -2 host factors extracted from public database (Virhosnet) or literature search. Table S6. Viral targets degrees HTLV_human_PPIs_MIMiX.txt PPIs experimental results - MIMiX standard. [file 1742-4690-9-26-S1.DOCX]

Additional Table S1 | List of viral ORFs and their grouping used in the homologous individual retest.

| Viral ORF | Virus name | Tax id | NCBI gene name | NCBI protein name | NCBI locus tag | Accession protein | Entrez gene id | group |
| --- | --- | --- | --- | --- | --- | --- | --- | --- |
| HTLV1_gag | HTLV1 | 11908 | gag | Pr55 | HTLV1gp3 | NP_057862 | 1491934 | gag_pol |
| HTLV1_pol | HTLV1 | 11908 | pol | Pr pol | HTLV1gp5 |  | 1491936 | gag_pol |
| HTLV1_rex | HTLV1 | 11908 | rex | p27 | HTLV1gp6 | NP_057863 | 1491937 | tax_rex |
| HTLV1_tax | HTLV1 | 11908 | tax | p40 | HTLV1gp7 | NP_057864 | 1491938 | tax_rex |
| HTLV1_env | HTLV1 | 11908 | env | gp46 SU | HTLV1gp8 | NP_057865 | 1491939 | env |
| HTLV1_p12 | HTLV1 | 11908 | p12 | P12 |  | DQ065793 |  | p30 |
| HTLV1_p30 | HTLV1 | 11908 | p30 | P30 |  | L08433 |  | p30 |
| HTLV1_p13 | HTLV1 | 11908 | p13 | P13 |  | L08433 |  | p30 |
| HTLV1_hbz | HTLV1 | 11908 | hbz | bZIP factor |  | ABB89742 |  | p30 |
| HTLV2_gag | HTLV2 | 11909 | HTLV2gp2 | gag polyprotein | HTLV2gp2 | NP_041002 | 1491944 | gag_pol |
| HTLV2_pol | HTLV2 | 11909 | HTLV2gp3 | pol polyprotein | HTLV2gp3 | NP_041003 | 1491943 | gag_pol |
| HTLV2_rex | HTLV2 | 11909 | HTLV2gp4 | rex 26 kD protein | HTLV2gp4 | NP_041004 | 1491945 | tax_rex |
| HTLV2_tax2 | HTLV2 | 11909 | HTLV2gp5 | tax protein | HTLV2gp5 | NP_041005 | 1491946 | tax_rex |
| HTLV2_env | HTLV2 | 11909 | HTLV2gp6 | env propeptide | HTLV2gp6 | NP_041006 | 1491942 | Env |
| HTLV2_aph2 | HTLV2 | 11909 |  |  |  |  |  |  |

Additional Table S2 | List of experimental results. Shows the orientation of the Y2H experiment in which the interactions was detected (human_y2h_side, Activation Domain AD, DNA Binding DB, or both). The relative activity as measured by the transactivation assay is shown for significant results with HTLV_LTR. MAPPIT results are shown in the last column.

| Viral ORF | Human entrez gene id | Human gene symbol | Human y2h side | HTLV LTR | MAPPIT |
| --- | --- | --- | --- | --- | --- |
| HTLV1_rex | 140735 | Dlc2 | AD | _ | 1 |
| HTLV1_rex | 1406 | CRX | AD | _ | 0 |
| HTLV1_rex | 51324 | SPG21 | DB | - | 0 |
| HTLV1_rex | 26150 | RIBC2 | DB | _ | 0 |
| HTLV1_rex | 60491 | NIF3L1 | DB | - | 0 |
| HTLV1_rex | 55165 | C10orf3 | AD | - | 0 |
| HTLV1_rex | 6672 | SP100 | AD | _ | 0 |
| HTLV1_rex | 9063 | PIAS2 | AD | _ | 0 |
| HTLV1_rex | 1856 | DVL2 | AD | _ | 0 |
| HTLV1_rex | 7329 | UBE2I | AD | _ | 0 |
| HTLV1_rex | 153657 | FLJ25439 | DB | _ | 1 |
| HTLV1_rex | 23636 | NUP62 | AD | _ | 0 |
| HTLV1_rex | 84186 | ZCCHC7 | AD | _ | 0 |
| HTLV1_rex | 8553 | BHLHB2 | AD | _ | 1 |
| HTLV1_rex | 84445 | LZTS2 | both | _ | 0 |
| HTLV1_rex | 391257 | LOC391257 | AD | _ | 0 |
| HTLV1_rex | 10615 | SPAG5 | AD | _ | 0 |
| HTLV1_rex | 222484 | LNX2 | both | _ | 0 |
| HTLV1_tax | 149473 | CCDC24 | DB | 2.88 | 0 |
| HTLV1_tax | 9827 | KIAA0258 | DB | _ | 1 |
| HTLV1_tax | 113802 | C1orf59 | DB | _ | 1 |
| HTLV1_tax | 84864 | MINA | AD | _ | 0 |
| HTLV1_tax | 64968 | MRPS6 | DB | 0.38 | 1 |
| HTLV1_tax | 79622 | C16orf33 | DB | _ | 0 |
| HTLV1_tax | 2189 | FANCG | DB | 3.48 | 1 |
| HTLV1_tax | 128653 | C20orf141 | DB | 2.80 | 0 |
| HTLV1_tax | 51367 | POP5 | DB | _ | 1 |
| HTLV1_tax | 51545 | ZNF581 | DB | _ | 0 |
| HTLV1_tax | 85359 | DGCR6L | DB | 0.24 | 1 |
| HTLV1_tax | 56998 | CTNNBIP1 | DB | _ | 0 |
| HTLV1_tax | 80110 | ZNF614 | DB | 5.24 | 1 |
| HTLV1_tax | 8738 | CRADD | DB | _ | 1 |
| HTLV1_tax | 50804 | MYEF2 | DB | 2.53 | 0 |
| HTLV1_tax | 10459 | MAD2L2 | DB | _ | 0 |
| HTLV1_tax | 51160 | VPS28 | DB | _ | 0 |
| HTLV1_tax | 54927 | CHCHD3 | DB | _ | 0 |
| HTLV1_tax | 3219 | HOXB9 | DB | 3.69 | 0 |
| HTLV1_tax | 5682 | PSMA1 | DB | _ | 0 |
| HTLV1_tax | 1406 | CRX | AD | _ | 0 |
| HTLV1_tax | 51324 | SPG21 | DB | 0.21 | 0 |
| HTLV1_tax | 26150 | RIBC2 | DB | _ | 1 |
| HTLV1_tax | 79873 | NUDT18 | DB | _ | 0 |
| HTLV1_tax | 80212 | FLJ22471 | DB | 2.25 | 1 |
| HTLV1_tax | 80125 | FLJ32855 | both | _ | 1 |
| HTLV1_tax | 60491 | NIF3L1 | DB | 0.39 | 1 |
| HTLV1_tax | 3866 | KRT15 | AD | _ | 1 |
| HTLV1_tax | 80254 | Cep63 | both | _ | 1 |
| HTLV1_tax | 9146 | HGS | both | _ | 0 |
| HTLV1_tax | 89885 | FATE1 | DB | 0.49 | 1 |
| HTLV1_tax | 7205 | TRIP6 | AD | _ | 0 |
| HTLV1_tax | 3853 | KRT6A | DB | _ | 0 |
| HTLV1_tax | 5992 | RFX4 | DB | _ | 1 |
| HTLV1_tax | 91661 | LOC91661 | DB | _ | 0 |
| HTLV1_tax | 51171 | DHRS10 | AD | 0.41 | 0 |
| HTLV1_tax | 23001 | WDFY3 | DB | _ | 1 |
| HTLV1_tax | 2118 | ETV4 | DB | _ | 1 |
| HTLV1_tax | 8697 | CDC23 | DB | _ | 0 |
| HTLV1_tax | 388818 | LOC388818 | AD | _ | 0 |
| HTLV1_tax | 84460 | ZMAT1 | DB | _ | 0 |
| HTLV1_tax | 4747 | NEFL | both | 0.45 | 0 |
| HTLV1_tax | 283385 | LOC283385 | DB | _ | 0 |
| HTLV1_tax | 153657 | FLJ25439 | DB | _ | 1 |
| HTLV1_tax | 83444 | ZNHIT4 | DB | _ | 0 |
| HTLV1_tax | 10106 | CTDSP2 | DB | _ | 1 |
| HTLV1_tax | 595101 | LOC595101 | DB | _ | 0 |
| HTLV1_tax | 3851 | KRT4 | DB | _ | 1 |
| HTLV1_tax | 64147 | KIF9 | DB | 0.37 | 0 |
| HTLV1_tax | 27434 | POLM | DB | _ | 1 |
| HTLV1_tax | 2130 | EWSR1 | DB | _ | 0 |
| HTLV1_tax | 3856 | KRT8 | DB | 0.24 | 1 |
| HTLV1_tax | 170954 | KIAA1949 | DB | 0.35 | 0 |
| HTLV1_tax | 4846 | NOS3 | DB | 0.45 | 1 |
| HTLV1_tax | 222484 | LNX2 | both | 0.28 | 0 |
| HTLV1_tax | 79165 | LENG1 | DB | 0.34 | 0 |
| HTLV1_tax | 64927 | TTC23 | DB | 0.21 | 0 |
| HTLV1_env | 81628 | TSC22D4 | DB | _ | 0 |
| HTLV1_env | 64773 | C20orf81 | DB | _ | 0 |
| HTLV1_hbz | 10962 | MLLT11 | AD | _ | 0 |
| HTLV1_hbz | 5015 | OTX2 | AD | _ | 0 |
| HTLV1_hbz | 9802 | DAZAP2 | AD | _ | 0 |
| HTLV1_hbz | 2495 | FTH1 | AD | _ | 1 |
| HTLV1_hbz | 6945 | MLX | DB | _ | 1 |
| HTLV1_hbz | 10921 | RNPS1 | DB | _ | 0 |
| HTLV1_hbz | 3232 | HOXD3 | AD | _ | 0 |
| HTLV1_hbz | 57120 | GOPC | DB | _ | 0 |
| HTLV1_hbz | 11143 | MYST2 | DB | _ | 0 |
| HTLV1_hbz | 5093 | PCBP1 | DB | _ | 0 |
| HTLV2_gag | 59349 | KLHL12 | AD | _ | 0 |
| HTLV2_gag | 7186 | TRAF2 | AD | _ | 0 |
| HTLV2_gag | 284252 | KCTD1 | AD | _ | 0 |
| HTLV2_gag | 222484 | LNX2 | AD | _ | 0 |
| HTLV2_gag | 1746 | DLX2 | AD | _ | 0 |
| HTLV2_gag | 24144 | TFIP11 | AD | _ | 0 |
| HTLV2_pol | 56159 | TEX11 | AD | _ | 0 |
| HTLV2_pol | 11030 | RBPMS | AD | _ | 0 |
| HTLV2_pol | 10174 | SORBS3 | AD | _ | 0 |
| HTLV2_pol | 3866 | KRT15 | AD | _ | 0 |
| HTLV2_pol | 4212 | MEIS2 | AD | _ | 1 |
| HTLV2_pol | 7681 | MKRN3 | AD | _ | 0 |
| HTLV2_pol | 147700 | KLC3 | AD | _ | 0 |
| HTLV2_pol | 24144 | TFIP11 | AD | _ | 0 |
| HTLV2_rex | 140735 | Dlc2 | AD | _ | 1 |
| HTLV2_rex | 1406 | CRX | AD | _ | 0 |
| HTLV2_rex | 51324 | SPG21 | DB | _ | 0 |
| HTLV2_rex | 60491 | NIF3L1 | DB | _ | 1 |
| HTLV2_rex | 6672 | SP100 | AD | _ | 0 |
| HTLV2_rex | 23636 | NUP62 | AD | _ | 0 |
| HTLV2_rex | 222484 | LNX2 | both | _ | 0 |
| HTLV2_tax2 | 140735 | Dlc2 | AD | _ | 0 |
| HTLV2_tax2 | 149473 | CCDC24 | DB | 2.88 | 0 |
| HTLV2_tax2 | 84970 | C1orf94 | AD | 0.41 | 0 |
| HTLV2_tax2 | 84864 | MINA | AD | _ | 0 |
| HTLV2_tax2 | 64968 | MRPS6 | DB | 0.38 | 1 |
| HTLV2_tax2 | 51545 | ZNF581 | DB | _ | 0 |
| HTLV2_tax2 | 85359 | DGCR6L | DB | 0.24 | 0 |
| HTLV2_tax2 | 79576 | NKAP | DB | _ | 0 |
| HTLV2_tax2 | 150483 | MGC27019 | DB | _ | 0 |
| HTLV2_tax2 | 80726 | KIAA1683 | DB | _ | 0 |
| HTLV2_tax2 | 7704 | ZBTB16 | DB | _ | 0 |
| HTLV2_tax2 | 84310 | MGC11257 | DB | _ | 0 |
| HTLV2_tax2 | 80110 | ZNF614 | DB | 5.24 | 0 |
| HTLV2_tax2 | 51329 | ARL6IP4 | DB | _ | 0 |
| HTLV2_tax2 | 51160 | VPS28 | DB | _ | 0 |
| HTLV2_tax2 | 9491 | PSMF1 | DB | 14.83 | 0 |
| HTLV2_tax2 | 1406 | CRX | AD | _ | 1 |
| HTLV2_tax2 | 51324 | SPG21 | DB | 0.21 | 1 |
| HTLV2_tax2 | 79873 | NUDT18 | DB | _ | 0 |
| HTLV2_tax2 | 80212 | FLJ22471 | DB | 2.25 | 0 |
| HTLV2_tax2 | 80125 | FLJ32855 | both | _ | 0 |
| HTLV2_tax2 | 60491 | NIF3L1 | DB | 0.39 | 0 |
| HTLV2_tax2 | 84766 | MGC4266 | DB | _ | 0 |
| HTLV2_tax2 | 3866 | KRT15 | AD | _ | 0 |
| HTLV2_tax2 | 6672 | SP100 | AD | _ | 0 |
| HTLV2_tax2 | 80254 | Cep63 | both | _ | 0 |
| HTLV2_tax2 | 8379 | MAD1L1 | DB | 0.20 | 0 |
| HTLV2_tax2 | 9146 | HGS | both | _ | 0 |
| HTLV2_tax2 | 89885 | FATE1 | DB | 0.49 | 0 |
| HTLV2_tax2 | 55093 | C8orf32 | DB | 0.26 | 0 |
| HTLV2_tax2 | 80321 | Cep70 | DB | _ | 0 |
| HTLV2_tax2 | 79571 | GCC1 | DB | 0.29 | 0 |
| HTLV2_tax2 | 8697 | CDC23 | DB | _ | 0 |
| HTLV2_tax2 | 388818 | LOC388818 | AD | _ | 1 |
| HTLV2_tax2 | 6660 | SOX5 | AD | _ | 1 |
| HTLV2_tax2 | 23636 | NUP62 | AD | _ | 0 |
| HTLV2_tax2 | 9513 | FXR2 | DB | 0.38 | 0 |
| HTLV2_tax2 | 595101 | LOC595101 | DB | _ | 0 |
| HTLV2_tax2 | 117178 | SSX2IP | DB | _ | 1 |
| HTLV2_tax2 | 9179 | AP4M1 | DB | _ | 0 |
| HTLV2_tax2 | 6517 | SLC2A4 | DB | _ | 0 |
| HTLV2_tax2 | 27434 | POLM | DB | _ | 0 |
| HTLV2_tax2 | 2130 | EWSR1 | DB | _ | 0 |
| HTLV2_tax2 | 8553 | BHLHB2 | AD | 0.20 | 0 |
| HTLV2_tax2 | 90480 | GADD45GIP1 | DB | 0.42 | 0 |
| HTLV2_tax2 | 1656 | DDX6 | DB | _ | 0 |
| HTLV2_tax2 | 84445 | LZTS2 | both | 0.23 | 1 |
| HTLV2_tax2 | 55216 | FLJ10726 | DB | 0.19 | 0 |
| HTLV2_tax2 | 222484 | LNX2 | both | 0.28 | 0 |
| HTLV2_env | 9443 | CRSP9 | DB | _ | 1 |
| HTLV2_aph2 | 50836 | TAS2R8 | AD | _ | _ |
| HTLV2_aph2 | 4779 | NFE2L1 | AD | _ | _ |
| HTLV2_aph2 | 137492 | VPS37A | AD | _ | _ |
| HTLV2_aph2 | 53371 | NUP54 | AD | _ | _ |
| HTLV2_aph2 | 200845 | KCTD6 | DB | _ | _ |
| HTLV2_aph2 | 7392 | USF2 | DB | _ | _ |
| HTLV2_aph2 | 58490 | RPRD1B | DB | _ | _ |
| HTLV2_aph2 | 388389 | CCDC103 | DB | _ | _ |

Additional Table S3 | Host factors regulating HTLV-1 LTR promoter activation by Tax.

| Gene Symbol | Entrez Gene Id | Viral Orfs | Transactivation |
| --- | --- | --- | --- |
| BHLHB2 | 8553 | HTLV2_tax2 | down |
| C1orf94 | 84970 | HTLV2_tax2 | down |
| C20orf141 | 128653 | HTLV1_tax | up |
| C8orf32 | 55093 | HTLV2_tax2 | down |
| CCDC24 | 149473 | HTLV2_tax2,HTLV1_tax | up |
| DGCR6L | 85359 | HTLV2_tax2,HTLV1_tax | down |
| DHRS10 | 51171 | HTLV1_tax | down |
| FANCG | 2189 | HTLV1_tax | up |
| FATE1 | 89885 | HTLV2_tax2,HTLV1_tax | down |
| FLJ10726 | 55216 | HTLV2_tax2 | down |
| FLJ22471 | 80212 | HTLV2_tax2,HTLV1_tax | up |
| FXR2 | 9513 | HTLV2_tax2 | down |
| GADD45GIP1 | 90480 | HTLV2_tax2 | down |
| GCC1 | 79571 | HTLV2_tax2 | down |
| HOXB9 | 3219 | HTLV1_tax | up |
| KIAA1949 | 170954 | HTLV1_tax | down |
| KIF9 | 64147 | HTLV1_tax | down |
| KRT8 | 3856 | HTLV1_tax | down |
| LENG1 | 79165 | HTLV1_tax | down |
| LNX2 | 222484 | HTLV2_tax2,HTLV1_tax | down |
| LZTS2 | 84445 | HTLV2_tax2 | down |
| MAD1L1 | 8379 | HTLV2_tax2 | down |
| MRPS6 | 64968 | HTLV2_tax2,HTLV1_tax | down |
| MYEF2 | 50804 | HTLV1_tax | up |
| NEFL | 4747 | HTLV1_tax | down |
| NIF3L1 | 60491 | HTLV2_tax2,HTLV1_tax | down |
| NOS3 | 4846 | HTLV1_tax | down |
| PSMF1 | 9491 | HTLV2_tax2 | up |
| SPG21 | 51324 | HTLV2_tax2,HTLV1_tax | down |
| TTC23 | 64927 | HTLV1_tax | down |
| ZNF614 | 80110 | HTLV2_tax2,HTLV1_tax | up |

Additional Table S4 | Human proteins interacting with Tax viral proteins.

| Gene Symbol | Entrez Gene Id | Viral Orfs |
| --- | --- | --- |
| C16orf33 | 79622 | HTLV1_tax |
| C1orf59 | 113802 | HTLV1_tax |
| C20orf141 | 128653 | HTLV1_tax |
| CHCHD3 | 54927 | HTLV1_tax |
| CRADD | 8738 | HTLV1_tax |
| CTDSP2 | 10106 | HTLV1_tax |
| CTNNBIP1 | 56998 | HTLV1_tax |
| DHRS10 | 51171 | HTLV1_tax |
| ETV4 | 2118 | HTLV1_tax |
| FANCG | 2189 | HTLV1_tax |
| FLJ25439 | 153657 | HTLV1_tax |
| HOXB9 | 3219 | HTLV1_tax |
| KIAA0258 | 9827 | HTLV1_tax |
| KIAA1949 | 170954 | HTLV1_tax |
| KIF9 | 64147 | HTLV1_tax |
| KRT4 | 3851 | HTLV1_tax |
| KRT6A | 3853 | HTLV1_tax |
| KRT8 | 3856 | HTLV1_tax |
| LENG1 | 79165 | HTLV1_tax |
| LOC283385 | 283385 | HTLV1_tax |
| LOC91661 | 91661 | HTLV1_tax |
| MAD2L2 | 10459 | HTLV1_tax |
| MYEF2 | 50804 | HTLV1_tax |
| NEFL | 4747 | HTLV1_tax |
| NOS3 | 4846 | HTLV1_tax |
| POP5 | 51367 | HTLV1_tax |
| PSMA1 | 5682 | HTLV1_tax |
| RFX4 | 5992 | HTLV1_tax |
| RIBC2 | 26150 | HTLV1_tax |
| TRIP6 | 7205 | HTLV1_tax |
| TTC23 | 64927 | HTLV1_tax |
| WDFY3 | 23001 | HTLV1_tax |
| ZMAT1 | 84460 | HTLV1_tax |
| ZNHIT4 | 83444 | HTLV1_tax |
| CCDC24 | 149473 | HTLV1_tax,HTLV2_tax2 |
| CDC23 | 8697 | HTLV1_tax,HTLV2_tax2 |
| Cep63 | 80254 | HTLV1_tax,HTLV2_tax2 |
| CRX | 1406 | HTLV1_tax,HTLV2_tax2 |
| DGCR6L | 85359 | HTLV1_tax,HTLV2_tax2 |
| EWSR1 | 2130 | HTLV1_tax,HTLV2_tax2 |
| FATE1 | 89885 | HTLV1_tax,HTLV2_tax2 |
| FLJ22471 | 80212 | HTLV1_tax,HTLV2_tax2 |
| FLJ32855 | 80125 | HTLV1_tax,HTLV2_tax2 |
| HGS | 9146 | HTLV1_tax,HTLV2_tax2 |
| KRT15 | 3866 | HTLV1_tax,HTLV2_tax2 |
| LNX2 | 222484 | HTLV1_tax,HTLV2_tax2 |
| LOC388818 | 388818 | HTLV1_tax,HTLV2_tax2 |
| LOC595101 | 595101 | HTLV1_tax,HTLV2_tax2 |
| MINA | 84864 | HTLV1_tax,HTLV2_tax2 |
| MRPS6 | 64968 | HTLV1_tax,HTLV2_tax2 |
| NIF3L1 | 60491 | HTLV1_tax,HTLV2_tax2 |
| NUDT18 | 79873 | HTLV1_tax,HTLV2_tax2 |
| POLM | 27434 | HTLV1_tax,HTLV2_tax2 |
| SPG21 | 51324 | HTLV1_tax,HTLV2_tax2 |
| VPS28 | 51160 | HTLV1_tax,HTLV2_tax2 |
| ZNF581 | 51545 | HTLV1_tax,HTLV2_tax2 |
| ZNF614 | 80110 | HTLV1_tax,HTLV2_tax2 |
| AP4M1 | 9179 | HTLV2_tax2 |
| ARL6IP4 | 51329 | HTLV2_tax2 |
| BHLHB2 | 8553 | HTLV2_tax2 |
| C1orf94 | 84970 | HTLV2_tax2 |
| C8orf32 | 55093 | HTLV2_tax2 |
| Cep70 | 80321 | HTLV2_tax2 |
| DDX6 | 1656 | HTLV2_tax2 |
| Dlc2 | 140735 | HTLV2_tax2 |
| FLJ10726 | 55216 | HTLV2_tax2 |
| FXR2 | 9513 | HTLV2_tax2 |
| GADD45GIP1 | 90480 | HTLV2_tax2 |
| GCC1 | 79571 | HTLV2_tax2 |
| KIAA1683 | 80726 | HTLV2_tax2 |
| LZTS2 | 84445 | HTLV2_tax2 |
| MAD1L1 | 8379 | HTLV2_tax2 |
| MGC11257 | 84310 | HTLV2_tax2 |
| MGC27019 | 150483 | HTLV2_tax2 |
| MGC4266 | 84766 | HTLV2_tax2 |
| NKAP | 79576 | HTLV2_tax2 |
| NUP62 | 23636 | HTLV2_tax2 |
| PSMF1 | 9491 | HTLV2_tax2 |
| SLC2A4 | 6517 | HTLV2_tax2 |
| SOX5 | 6660 | HTLV2_tax2 |
| SP100 | 6672 | HTLV2_tax2 |
| SSX2IP | 117178 | HTLV2_tax2 |
| ZBTB16 | 7704 | HTLV2_tax2 |

Additional Table S5 | List of HTLV-1 and -2 host factors extracted from public database (Virhosnet) or literature search. Sources indicate the pubmed id and/or virhostnet origin.

| viral_orf | human_geneid | human_genesymbol | sources |
| --- | --- | --- | --- |
| HTLV1_env | 5127 | CDK16 | virhostnet |
| HTLV1_env | 1739 | DLG1 | 15286176,virhostnet |
| HTLV1_env | 9787 | DLGAP5 | virhostnet |
| HTLV1_env | 125113 | KRT222 | virhostnet |
| HTLV1_env | 8829 | NRP1 | 16809290 |
| HTLV1_env | 387 | RHOA | virhostnet |
| HTLV1_env | 6513 | SLC2A1 | 14622599 |
| HTLV1_env | 6605 | SMARCE1 | virhostnet |
| HTLV1_hbz | 466 | ATF1 | 17151132 |
| HTLV1_hbz | 1385 | CREB1 | 17151132 |
| HTLV1_hbz | 1387 | CREBBP | 18599479 |
| HTLV1_hbz | 1390 | CREM | 17151132 |
| HTLV1_hbz | 3725 | JUN | 15044019 |
| HTLV1_hbz | 3726 | JUNB | 15044019 |
| HTLV1_hbz | 3727 | JUND | 15044019 |
| HTLV1_rex | 3267 | AGFG1 | virhostnet |
| HTLV1_rex | 8021 | NUP214 | virhostnet |
| HTLV1_rex | 4928 | NUP98 | virhostnet |
| HTLV1_rex | 84268 | RPAIN | virhostnet |
| HTLV1_rex | 7514 | XPO1 | 14612415 |
| HTLV1_tax | 59 | ACTA2 | 14530271,virhostnet |
| HTLV1_tax | 86 | ACTL6A | 14530271,virhostnet |
| HTLV1_tax | 207 | AKT1 | 14530271 |
| HTLV1_tax | 11199 | ANXA10 | 14530271 |
| HTLV1_tax | 309 | ANXA6 | virhostnet |
| HTLV1_tax | 466 | ATF1 | 8628284 |
| HTLV1_tax | 1386 | ATF2 | 8407959,virhostnet |
| HTLV1_tax | 467 | ATF3 | 8007991 |
| HTLV1_tax | 468 | ATF4 | 9190894,virhostnet |
| HTLV1_tax | 22809 | ATF5 | 15890932 |
| HTLV1_tax | 9564 | BCAR1 | 15592516 |
| HTLV1_tax | 811 | CALR | 17395420,virhostnet |
| HTLV1_tax | 10498 | CARM1 | 17005681,virhostnet |
| HTLV1_tax | 595 | CCND1 | 9584203 |
| HTLV1_tax | 894 | CCND2 | 11314046,virhostnet |
| HTLV1_tax | 896 | CCND3 | 9584203,virhostnet |
| HTLV1_tax | 904 | CCNT1 | 17686863 |
| HTLV1_tax | 991 | CDC20 | 15623561 |
| HTLV1_tax | 8697 | CDC23 | 15623561 |
| HTLV1_tax | 998 | CDC42 | 14530271,virhostnet |
| HTLV1_tax | 1017 | CDK2 | virhostnet |
| HTLV1_tax | 1019 | CDK4 | 11971966,virhostnet |
| HTLV1_tax | 1021 | CDK6 | 11971966,virhostnet |
| HTLV1_tax | 1029 | CDKN2A | 8612584 |
| HTLV1_tax | 1030 | CDKN2B | 10388662 |
| HTLV1_tax | 1051 | CEBPB | 9376596,virhostnet |
| HTLV1_tax | 1111 | CHEK1 | 15107832,virhostnet |
| HTLV1_tax | 11200 | CHEK2 | 12842897,virhostnet |
| HTLV1_tax | 1147 | CHUK | 9632633 |
| HTLV1_tax | 1315 | COPB1 | 17897946 |
| HTLV1_tax | 1385 | CREB1 | 1386673,virhostnet |
| HTLV1_tax | 1387 | CREBBP | 8602268,virhostnet |
| HTLV1_tax | 1390 | CREM | 8628284 |
| HTLV1_tax | 23373 | CRTC1 | 16809310 |
| HTLV1_tax | 200186 | CRTC2 | 16809310 |
| HTLV1_tax | 64784 | CRTC3 | 15466468 |
| HTLV1_tax | 1739 | DLG1 | 9192623,virhostnet |
| HTLV1_tax | 1742 | DLG4 | 9482110,virhostnet |
| HTLV1_tax | 9093 | DNAJA3 | 11719219 |
| HTLV1_tax | 1958 | EGR1 | 9341193 |
| HTLV1_tax | 3646 | EIF3E | 8688078,virhostnet |
| HTLV1_tax | 2002 | ELK1 | 11070040 |
| HTLV1_tax | 2005 | ELK4 | 11070040 |
| HTLV1_tax | 2113 | ETS1 | 9030555,virhostnet |
| HTLV1_tax | 2783 | GNB2 | 16990599 |
| HTLV1_tax | 2874 | GPS2 | 9325311,virhostnet |
| HTLV1_tax | 2934 | GSN | 14530271,virhostnet |
| HTLV1_tax | 2957 | GTF2A1 | 8756622,virhostnet |
| HTLV1_tax | 3065 | HDAC1 | 12370815,virhostnet |
| HTLV1_tax | 3551 | IKBKB | 9632633 |
| HTLV1_tax | 8517 | IKBKG | 10364167,8170951,virhostnet |
| HTLV1_tax | 3603 | IL16 | 12620798 |
| HTLV1_tax | 9118 | INA | 9435256,virhostnet |
| HTLV1_tax | 9682 | JMJD2A | 8688078 |
| HTLV1_tax | 8850 | KAT2B | 10567539,virhostnet |
| HTLV1_tax | 3856 | KRT8 | 11725133 |
| HTLV1_tax | 8825 | LIN7A | 9482110 |
| HTLV1_tax | 8379 | MAD1L1 | 9546394,virhostnet |
| HTLV1_tax | 260425 | MAGI3 | 15003862 |
| HTLV1_tax | 4214 | MAP3K1 | 9630230 |
| HTLV1_tax | 6885 | MAP3K7 | 14530271 |
| HTLV1_tax | 23118 | MAP3K7IP2 | 17986383 |
| HTLV1_tax | 8932 | MBD2 | 15674330,virhostnet |
| HTLV1_tax | 4488 | MSX2 | 15970589 |
| HTLV1_tax | 9612 | NCOR2 | 12642864 |
| HTLV1_tax | 4739 | NEDD9 | 15592516,virhostnet |
| HTLV1_tax | 4790 | NFKB1 | 8361755,8692272 |
| HTLV1_tax | 4791 | NFKB2 | 7856081,8289813 |
| HTLV1_tax | 4792 | NFKBIA | 7700645 |
| HTLV1_tax | 4801 | NFYB | 9032250 |
| HTLV1_tax | 4899 | NRF1 | 10381170,virhostnet |
| HTLV1_tax | 23636 | NUP2 | 17344183 |
| HTLV1_tax | 5063 | PAK3 | virhostnet |
| HTLV1_tax | 5295 | PIK3R1 | 16436385 |
| HTLV1_tax | 5515 | PPP2CA | 12419799 |
| HTLV1_tax | 5516 | PPP2CB | 12419799 |
| HTLV1_tax | 5524 | PPP2R4 | 12419799 |
| HTLV1_tax | 5685 | PSMA4 | 8692272 |
| HTLV1_tax | 5692 | PSMB4 | 8692272 |
| HTLV1_tax | 5708 | PSMD2 | 17897946 |
| HTLV1_tax | 5879 | RAC1 | virhostnet |
| HTLV1_tax | 5888 | RAD51 | 14530271,virhostnet |
| HTLV1_tax | 5897 | RAG2 | virhostnet |
| HTLV1_tax | 5902 | RANBP1 | 16365316 |
| HTLV1_tax | 5903 | RANBP2 | 17897946 |
| HTLV1_tax | 5922 | RASA2 | 14530271,virhostnet |
| HTLV1_tax | 5925 | RB1 | 15580311 |
| HTLV1_tax | 5966 | REL | 7936632 |
| HTLV1_tax | 5970 | RELA | 7936632 |
| HTLV1_tax | 387 | RHOA | 14530271 |
| HTLV1_tax | 6128 | RPL6 | 12007002,virhostnet |
| HTLV1_tax | 9522 | SCAMP1 | 17897946 |
| HTLV1_tax | 10066 | SCAMP2 | 17897946 |
| HTLV1_tax | 23513 | SCRIB | 17855372 |
| HTLV1_tax | 10484 | SEC23A | 17897946 |
| HTLV1_tax | 10483 | SEC23B | 17897946 |
| HTLV1_tax | 6427 | SFRS2 | 8709263 |
| HTLV1_tax | 4087 | SMAD2 | 11264182 |
| HTLV1_tax | 4088 | SMAD3 | 11264182 |
| HTLV1_tax | 4089 | SMAD4 | 11264182 |
| HTLV1_tax | 6597 | SMARCA4 | 14530271,virhostnet |
| HTLV1_tax | 6599 | SMARCC1 | 14530271,virhostnet |
| HTLV1_tax | 6605 | SMARCE1 | 14530271 |
| HTLV1_tax | 8773 | SNAP23 | 17897946 |
| HTLV1_tax | 6641 | SNTB1 | 9482110,virhostnet |
| HTLV1_tax | 6645 | SNTB2 | 9482110 |
| HTLV1_tax | 6667 | SP1 | 9030555,virhostnet |
| HTLV1_tax | 6688 | SPI1 | 9376596 |
| HTLV1_tax | 6722 | SRF | 1427072 |
| HTLV1_tax | 23524 | SRRM2 | virhostnet |
| HTLV1_tax | 6839 | SUV39H1 | 16409643 |
| HTLV1_tax | 6882 | TAF11 | 9108034 |
| HTLV1_tax | 6895 | TARBP2 | 9060615,virhostnet |
| HTLV1_tax | 8887 | TAX1BP1 | 17283140,19131965,virhostnet |
| HTLV1_tax | 30851 | TAX1BP3 | 10940294 |
| HTLV1_tax | 6908 | TBP | 8223437 |
| HTLV1_tax | 7150 | TOP1 | 10792988,virhostnet |
| HTLV1_tax | 7334 | UBE2N | 17942533 |
| HTLV1_tax | 55697 | VAC14 | 16767081 |
| HTLV1_tax | 7494 | XBP1 | 18287238 |
| HTLV1_tax | 7514 | XPO1 | 16775353 |
| HTLV1_tax | 7538 | ZFP36 | 14679154,virhostnet |
| HTLV2_env | 1739 | DLG1 | virhostnet |
| HTLV2_env | 9787 | DLGAP5 | virhostnet |
| HTLV2_env | 8829 | NRP1 | 16809290 |
| HTLV2_env | 6513 | SLC2A1 | 14622599 |
| HTLV2_aph2 | 1385 | CREB1 | 19602711 |

Additional Table S7 | Degrees of HTLV human targets in the human-human interactome. k rual indicates the degree k in the network from Rual et al., Nature 2005; k lci indicates the degree k in the network extracted from several databases as described in the main text

| Human entrez gene id | Human gene symbol | Number of viral interactors | Viral interactors | k rual | k lci |
| --- | --- | --- | --- | --- | --- |
| 7186 | TRAF2 | 1 | HTLV2_gag | 70 | 169 |
| 2130 | EWSR1 | 2 | HTLV1_tax,HTLV2_tax2 | 94 | 119 |
| 7329 | UBE2I | 1 | HTLV1_rex | 21 | 99 |
| 7704 | ZBTB16 | 1 | HTLV2_tax2 | 12 | 55 |
| 9513 | FXR2 | 1 | HTLV2_tax2 | 50 | 54 |
| 1856 | DVL2 | 1 | HTLV1_rex | 36 | 52 |
| 3866 | KRT15 | 3 | HTLV2_tax2,HTLV2_pol,HTLV1_tax | 50 | 50 |
| 11030 | RBPMS | 1 | HTLV2_pol | 45 | 48 |
| 9146 | HGS | 2 | HTLV1_tax,HTLV2_tax2 | 21 | 46 |
| 9802 | DAZAP2 | 1 | HTLV1_hbz | 34 | 43 |
| 7205 | TRIP6 | 1 | HTLV1_tax | 14 | 35 |
| 60491 | NIF3L1 | 4 | HTLV1_tax,HTLV2_tax2,HTLV1_rex,HTLV2_rex | 30 | 35 |
| 81628 | TSC22D4 | 1 | HTLV1_env | 27 | 33 |
| 11143 | MYST2 | 1 | HTLV1_hbz | 10 | 31 |
| 79873 | NUDT18 | 2 | HTLV1_tax,HTLV2_tax2 | 29 | 30 |
| 6517 | SLC2A4 | 1 | HTLV2_tax2 |  | 29 |
| 5093 | PCBP1 | 1 | HTLV1_hbz |  | 26 |
| 23636 | NUP62 | 3 | HTLV1_rex,HTLV2_rex,HTLV2_tax2 | 6 | 24 |
| 56159 | TEX11 | 1 | HTLV2_pol | 23 | 24 |
| 3856 | KRT8 | 1 | HTLV1_tax |  | 21 |
| 80125 | FLJ32855 | 2 | HTLV2_tax2,HTLV1_tax | 20 | 21 |
| 5682 | PSMA1 | 1 | HTLV1_tax | 11 | 20 |
| 26150 | RIBC2 | 2 | HTLV1_rex,HTLV1_tax | 20 | 20 |
| 10174 | SORBS3 | 1 | HTLV2_pol | 13 | 18 |
| 55093 | C8orf32 | 1 | HTLV2_tax2 | 17 | 18 |
| 57120 | GOPC | 1 | HTLV1_hbz | 1 | 18 |
| 4747 | NEFL | 1 | HTLV1_tax |  | 17 |
| 8379 | MAD1L1 | 1 | HTLV2_tax2 | 9 | 17 |
| 80321 | Cep70 | 1 | HTLV2_tax2 | 16 | 17 |
| 9063 | PIAS2 | 1 | HTLV1_rex | 2 | 16 |
| 10921 | RNPS1 | 1 | HTLV1_hbz | 2 | 16 |
| 4846 | NOS3 | 1 | HTLV1_tax |  | 15 |
| 9491 | PSMF1 | 1 | HTLV2_tax2 | 13 | 15 |
| 59349 | KLHL12 | 1 | HTLV2_gag | 13 | 14 |
| 80254 | Cep63 | 2 | HTLV2_tax2,HTLV1_tax | 3 | 14 |
| 2189 | FANCG | 1 | HTLV1_tax | 6 | 13 |
| 51324 | SPG21 | 4 | HTLV1_tax,HTLV2_rex,HTLV1_rex,HTLV2_tax2 | 10 | 11 |
| 83444 | ZNHIT4 | 1 | HTLV1_tax |  | 11 |
| 84445 | LZTS2 | 2 | HTLV2_tax2,HTLV1_rex | 8 | 11 |
| 140735 | Dlc2 | 3 | HTLV1_rex,HTLV2_tax2,HTLV2_rex | 7 | 11 |
| 2495 | FTH1 | 1 | HTLV1_hbz | 2 | 10 |
| 79571 | GCC1 | 1 | HTLV2_tax2 | 8 | 10 |
| 8553 | BHLHB2 | 2 | HTLV2_tax2,HTLV1_rex |  | 9 |
| 9443 | CRSP9 | 1 | HTLV2_env | 1 | 9 |
| 10459 | MAD2L2 | 1 | HTLV1_tax |  | 9 |
| 4779 | NFE2L1 | 1 | HTLV2_aph2 |  | 9 |
| 3219 | HOXB9 | 1 | HTLV1_tax | 4 | 8 |
| 8738 | CRADD | 1 | HTLV1_tax |  | 8 |
| 51160 | VPS28 | 2 | HTLV1_tax,HTLV2_tax2 | 1 | 8 |
| 51545 | ZNF581 | 2 | HTLV2_tax2,HTLV1_tax | 8 | 8 |
| 7392 | USF2 | 1 | HTLV2_aph2 |  | 8 |
| 3853 | KRT6A | 1 | HTLV1_tax | 4 | 7 |
| 6672 | SP100 | 3 | HTLV2_rex,HTLV1_rex,HTLV2_tax2 | 1 | 7 |
| 55165 | C10orf3 | 1 | HTLV1_rex | 2 | 7 |
| 85359 | DGCR6L | 2 | HTLV1_tax,HTLV2_tax2 | 7 | 7 |
| 147700 | KLC3 | 1 | HTLV2_pol |  | 7 |
| 1656 | DDX6 | 1 | HTLV2_tax2 |  | 6 |
| 6945 | MLX | 1 | HTLV1_hbz | 1 | 6 |
| 80726 | KIAA1683 | 1 | HTLV2_tax2 | 6 | 6 |
| 84970 | C1orf94 | 1 | HTLV2_tax2 | 4 | 6 |
| 117178 | SSX2IP | 1 | HTLV2_tax2 |  | 6 |
| 6660 | SOX5 | 1 | HTLV2_tax2 |  | 5 |
| 8697 | CDC23 | 2 | HTLV1_tax,HTLV2_tax2 | 3 | 5 |
| 10615 | SPAG5 | 1 | HTLV1_rex |  | 5 |
| 51367 | POP5 | 1 | HTLV1_tax |  | 5 |
| 89885 | FATE1 | 2 | HTLV2_tax2,HTLV1_tax | 4 | 5 |
| 90480 | GADD45GIP1 | 1 | HTLV2_tax2 |  | 5 |
| 53371 | NUP54 | 1 | HTLV2_aph2 | 3 | 5 |
| 1406 | CRX | 4 | HTLV1_rex,HTLV2_tax2,HTLV1_tax,HTLV2_rex | 2 | 4 |
| 5015 | OTX2 | 1 | HTLV1_hbz |  | 4 |
| 9179 | AP4M1 | 1 | HTLV2_tax2 |  | 4 |
| 51171 | DHRS10 | 1 | HTLV1_tax | 4 | 4 |
| 56998 | CTNNBIP1 | 1 | HTLV1_tax | 2 | 4 |
| 1746 | DLX2 | 1 | HTLV2_gag |  | 3 |
| 5992 | RFX4 | 1 | HTLV1_tax |  | 3 |
| 79576 | NKAP | 1 | HTLV2_tax2 | 3 | 3 |
| 137492 | VPS37A | 1 | HTLV2_aph2 |  | 3 |
| 2118 | ETV4 | 1 | HTLV1_tax |  | 2 |
| 24144 | TFIP11 | 2 | HTLV2_gag,HTLV2_pol |  | 2 |
| 54927 | CHCHD3 | 1 | HTLV1_tax | 2 | 2 |
| 64968 | MRPS6 | 2 | HTLV1_tax,HTLV2_tax2 |  | 2 |
| 84766 | MGC4266 | 1 | HTLV2_tax2 | 1 | 2 |
| 84864 | MINA | 2 | HTLV1_tax,HTLV2_tax2 | 1 | 2 |
| 150483 | MGC27019 | 1 | HTLV2_tax2 | 2 | 2 |
| 222484 | LNX2 | 5 | HTLV2_tax2,HTLV1_tax,HTLV2_gag,HTLV1_rex,HTLV2_rex |  | 2 |
| 27434 | POLM | 2 | HTLV1_tax,HTLV2_tax2 |  | 1 |
| 51329 | ARL6IP4 | 1 | HTLV2_tax2 |  | 1 |
| 64773 | C20orf81 | 1 | HTLV1_env | 1 | 1 |
| 79165 | LENG1 | 1 | HTLV1_tax |  | 1 |
| 80212 | FLJ22471 | 2 | HTLV1_tax,HTLV2_tax2 |  | 1 |
| 84186 | ZCCHC7 | 1 | HTLV1_rex |  | 1 |
| 84310 | MGC11257 | 1 | HTLV2_tax2 | 1 | 1 |
| 149473 | CCDC24 | 2 | HTLV1_tax,HTLV2_tax2 |  | 1 |
| 58490 | RPRD1B | 1 | HTLV2_aph2 | 1 | 1 |
| 3232 | HOXD3 | 1 | HTLV1_hbz |  |  |
| 3851 | KRT4 | 1 | HTLV1_tax |  |  |
| 4212 | MEIS2 | 1 | HTLV2_pol |  |  |
| 7681 | MKRN3 | 1 | HTLV2_pol |  |  |
| 9827 | KIAA0258 | 1 | HTLV1_tax |  |  |
| 10106 | CTDSP2 | 1 | HTLV1_tax |  |  |
| 10962 | MLLT11 | 1 | HTLV1_hbz |  |  |
| 23001 | WDFY3 | 1 | HTLV1_tax |  |  |
| 50804 | MYEF2 | 1 | HTLV1_tax |  |  |
| 55216 | FLJ10726 | 1 | HTLV2_tax2 |  |  |
| 64147 | KIF9 | 1 | HTLV1_tax |  |  |
| 64927 | TTC23 | 1 | HTLV1_tax |  |  |
| 79622 | C16orf33 | 1 | HTLV1_tax |  |  |
| 80110 | ZNF614 | 2 | HTLV2_tax2,HTLV1_tax |  |  |
| 84460 | ZMAT1 | 1 | HTLV1_tax |  |  |
| 91661 | LOC91661 | 1 | HTLV1_tax |  |  |
| 113802 | C1orf59 | 1 | HTLV1_tax |  |  |
| 128653 | C20orf141 | 1 | HTLV1_tax |  |  |
| 153657 | FLJ25439 | 2 | HTLV1_rex,HTLV1_tax |  |  |
| 170954 | KIAA1949 | 1 | HTLV1_tax |  |  |
| 283385 | LOC283385 | 1 | HTLV1_tax |  |  |
| 284252 | KCTD1 | 1 | HTLV2_gag |  |  |
| 388818 | LOC388818 | 2 | HTLV2_tax2,HTLV1_tax |  |  |
| 391257 | LOC391257 | 1 | HTLV1_rex |  |  |
| 595101 | LOC595101 | 2 | HTLV1_tax,HTLV2_tax2 |  |  |
| 50836 | TAS2R8 | 1 | HTLV2_aph2 |  |  |
| 200845 | KCTD6 | 1 | HTLV2_aph2 |  |  |
| 388389 | CCDC103 | 1 | HTLV2_aph2 |  |  |
